# Defect‐Engineered Bulk Conversion Anodes for Fast and Temperature‐Adaptive Na^+^ Storage

**DOI:** 10.1002/advs.202515276

**Published:** 2025-10-17

**Authors:** Yanli Zhou, Ao Xu, Zhiqi Li, Yifei Wang, Jiawen Yan, Fuyi Jiang, Wei Liu, Xin Gu, Huan Pang, Jian Yang

**Affiliations:** ^1^ Shandong Key Laboratory of Advanced Structural Materials Genome Engineering School of Environmental and Material Engineering Yantai University Yantai Shandong 264005 China; ^2^ Key Laboratory of Colloid and Interface Chemistry Ministry of Education School of Chemistry and Chemical Engineering Shandong University Jinan 250100 China; ^3^ Shandong Key Laboratory of Advanced Electrochemical Energy Storage Technologies College of New Energy State Key Laboratory of Heavy Oil Processing China University of Petroleum (East China) Qingdao Shandong 266580 China; ^4^ School of Chemistry and Chemical Engineering Yangzhou University Yangzhou Jiangsu 225009 China

**Keywords:** bulk‐Fe_7_Se_8‐x_, carbon‐free, defects engineering, sodium‐ion batteries, temperature adaptability

## Abstract

Conversion‐based metal sulfides/selenides anodes are promising for sodium‐ion batteries (SIBs) owing to their high capacities and good electronic conductivity. However, their large volumetric variation during cycling often leads to rapid capacity decay, hindering commercial applications. Meanwhile, developing a low‐cost and efficient synthesis strategy to enhance sodium storage remains challenging. Herein, defect engineering is introduced in bulk Fe_7_Se_8_ (bulk‐Fe_7_Se_8‐x_) to fabricate a carbon‐free and defect‐rich anode. The optimized bulk‐Fe_7_Se_8‐x_ realizes remarkable fast‐charging performance and good temperature adaptability, achieving excellent cyclic stability (384 mAh g^−1^ after 1300 cycles at 5 A g^−1^ and 25 °C, 170.6 mAh g^−1^ after 1000 cycles at 2 A g^−1^ and 0 °C, and 371.7 mAh g^−1^ after 500 cycles at 5 A g^−1^ and 40 °C) and ultrahigh rate capability (up to 40 A g^−1^), significantly surpassing reported Fe_7_Se_8_ anodes. Reaction mechanism and reaction kinetics are elucidated through in/ex situ characterization, kinetics analysis, and DFT calculations. Furthermore, the exploration of full cells demonstrates their potential application of bulk‐Fe_7_Se_8‐x_ in SIBs. The universal strategy is also successfully applied to synthesize the high‐performance, defect‐rich bulk‐Fe_7_S_8‐x_ and bulk‐CoSe_2‐x_, which provides an effective approach for other conversion‐based anode materials.

## Graphical abstract

Defect‐engineering has been rationally applied in bulk conversion anodes for sodium ion batteries. The typical carbon‐free/defect‐rich bulk‐Fe_7_Se_8‐x_ realizes extraordinary fast and temperature‐adaptive sodium ion storage. Various *in*/*ex situ* experimental characterizations and DFT calculations verify the peculiar conversion‐type reaction mechanism and fast reaction kinetics. Such a universal synthesis strategy provides a powerful route for the performance optimization of other conversion anodes.

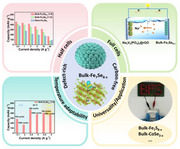

## 1 Introduction

The growing demand for renewable energy utilization and increasing environmental concerns have intensified the need for efficient and scalable energy storage technologies.^[^ ^1^, ^2^ ^]^ In contrast with lithium, sodium is more abundant and cost‐effective on Earth. Meanwhile, sodium‐ion batteries (SIBs) work on a similar principle to lithium‐ion batteries (LIBs), making them a promising alternative. However, graphite, a widely adopted anode for LIBs, exhibits limited sodium storage capacity due to thermodynamic constraints, hindering the advancement of SIBs.^[^ ^3^, ^4^, ^5^ ^]^ Thus, it is imperative to develop low‐cost and high‐performance anode materials for SIBs.

Among various anode materials for SIBs, transition metal chalcogenides have attracted great interest owing to their noticeable advantages, including their high theoretical capacities, suitable redox potentials, and natural abundance.^[^ ^6^, ^7^ ^]^ Fe_7_Se_8_, which operates via a typical conversion mechanism, is particularly promising owing to its theoretical capacity of 419 mAh g^−1^, good electronic conductivity, and low synthesis cost. However, its giant volume expansion during cycling induces substantial stress, resulting in electrode pulverization, detachment from the current collector, and loss of electrical contact. Moreover, the solid electrolyte interfaces (SEI) formed in ester‐based electrolytes tend to be unstable. Repeated SEI fracture and reformation consume both active sodium ions and electrolyte, resulting in rapid capacity decay.

To address these issues, intense efforts have been devoted to improving the sodium storage performance of Fe_7_Se_8_ for SIBs. A widely adopted strategy involves constructing nanostructures combined with carbon modification. Nanostructuring shortens the migration distance of sodium ions and provides abundant electroactive sites.^[^ ^8^ ^]^ Carbon incorporation enhances overall electronic conductivity and partially alleviates volumetric expansion. For instance, N‐doped carbon modified yolk‐shell Fe_7_Se_8_ nanoboxes prepared via two‐step methods, exhibited an excellent rate capability (316 mAh g^−1^ at 5 A g^−1^).^[^ ^9^ ^]^ Similarly, nano‐sized Fe_7_Se_8_ nano‐particles encapsulated in N‐doped carbon nanofibers (Fe_7_Se_8_/N‐CNFs) displayed improved rate performance (286 mAh g^−1^ at 20 A g^−1^).^[^ ^10^ ^]^ Despite these advances, the practical application of Fe_7_Se_8_‐based anodes in SIBs remains challenging.^[^ ^11^, ^12^, ^13^ ^]^ First, current synthesis strategies for Fe_7_Se_8_‐based electrodes often involve multi‐step reactions and harsh conditions, increasing the production cost and complicating scale‐up. Second, although conductive carbon additives enhance electrical conductivity, they lower overall capacity, reducing energy density. Third, while nanostructuring (e.g., hollow or porous architectures) can mitigate volume changes, it often lowers the tap density, thereby limiting volumetric energy density. Therefore, developing low‐cost and high‐performance Fe_7_Se_8_‐based electrodes through scale processes is still a huge challenge.

Herein, we demonstrate a defect‐assisted strategy to enhance the sodium storage performance of bulk transition metal chalcogenides, using Fe_7_Se_8_ as an example. Bulk‐Fe_7_Se_8_ anodes with different defect concentrations were prepared by varying the ratio of Fe/Se in a low‐cost calcination process. When coupled with NaPF_6_ in an ether‐based electrolyte, the carbon‐free bulk‐Fe_7_Se_8‐x_ (Fe:Se = 1:8) with moderate selenium defects exhibits a stable electrode interface, excellent sodium storage properties, and wide temperature adaptability. Specifically, it delivers a high capacity of 447.3 mAh g^−1^ at 0.5 A g^−1^ (25 °C), ultrahigh rate capability up to 40 A g^−1^ (25 and 40 °C) and remarkable cyclic stability (384 mAh g^−1^ after 1300 cycles at 5 A g^−1^ and 25 °C), outperforming both controls (e.g., highly defective bulk‐Fe_7_Se_8‐x_ (Fe:Se = 1:4), defect‐free bulk‐Fe_7_Se_8_ (1:12), carbon‐rich/defect‐free nano‐Fe_7_Se_8_) and previously reported Fe_7_Se_8_‐based anodes. The underlying mechanism was elucidated through experimental techniques and theoretical calculations. The full cells of Na_3_V_2_(PO_4_)_3_@rGO//bulk‐Fe_7_Se_8‐x_ successfully powered LEDs, demonstrating the application potential of carbon‐free bulk‐Fe_7_Se_8‐x_ in SIBs. The present synthesis reduces synthesis steps and production cost, minimizes the carbon usage, and improves specific capacities. Notably, this strategy was successfully extended to prepare high‐performance bulk‐Fe_7_S_8‐x_ and bulk‐CoSe_2‐x_, verifying the universality for conversion‐type anodes.

## 2 Results and Discussion

**Figure 1:**
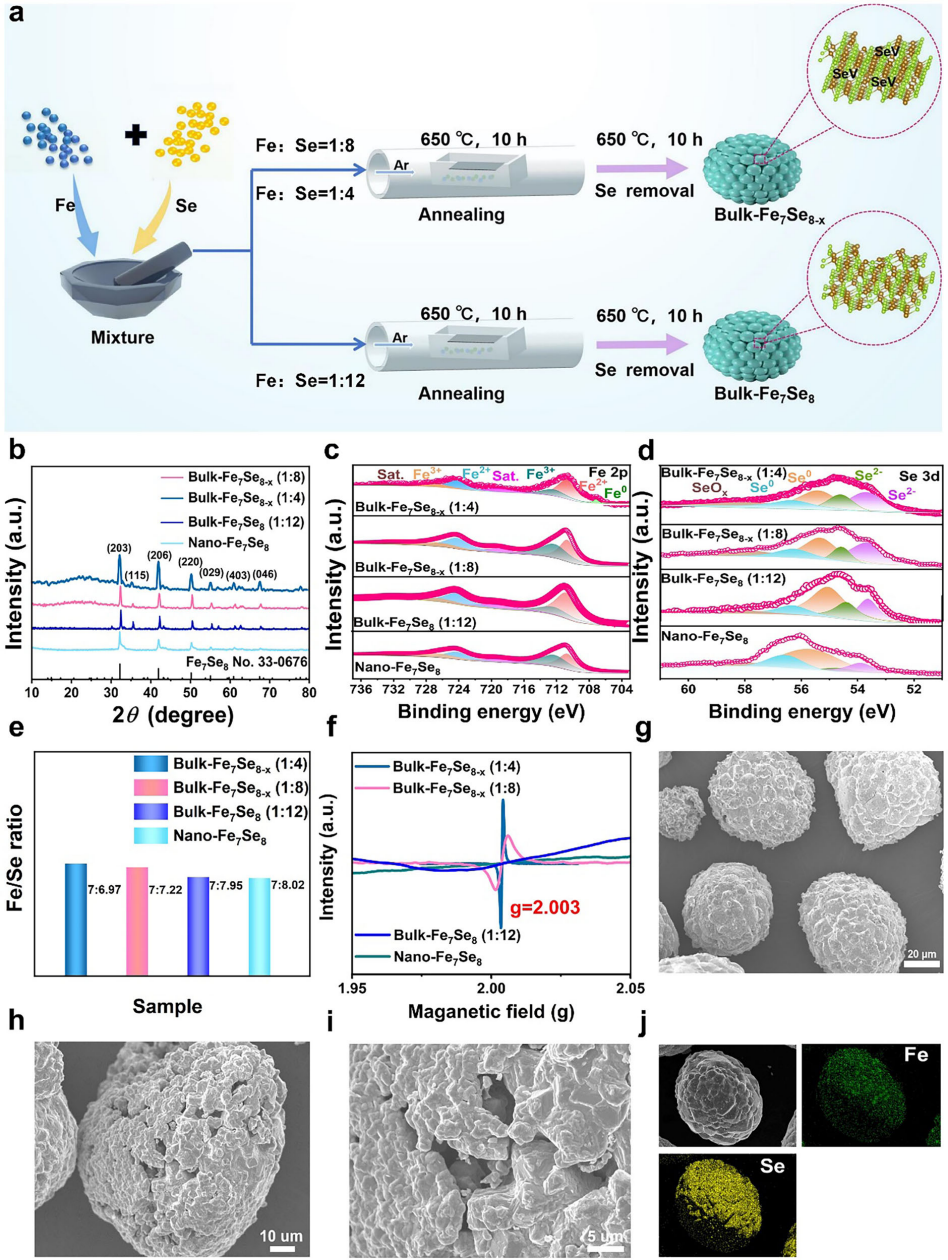
Structure and morphology characterizations. a) Schematic illustration of synthesis strategy, b) XRD patterns, XPS spectra of c) Fe 2p, and d) Se 3d, e) atomic ratios of Fe/Se, f) EPR spectra of four samples, g–i) SEM images, and j) element mapping of bulk‐Fe_7_Se_8‐x_ (1:8).

**Figure** **1**a illustrates the defect‐engineered synthesis strategy. Micro‐sized iron powders reacted with selenium at 650 °C in Ar/H_2_ to produce bulk‐Fe_7_Se_8_. By varying the Fe/Se molar ratio (1:4, 1:8 or 1:12), defect‐rich bulk‐Fe_7_Se_8‐x_ or defect‐free bulk‐Fe_7_Se_8_ was obtained via one‐step solid‐phase reaction. The XRD patterns of bulk‐Fe_7_Se_8‐x_ (Figure 1b; Table S1, Supporting Information) indicate that all the strong diffraction peaks are well indexed as the hexagonal Fe_7_Se_8_ (JCPDS No. 33–0676), consistent with bulk‐Fe_7_Se_8_ and nano‐Fe_7_Se_8_. The XPS spectra suggest the existence of Fe and Se in all samples (Figure S1, Supporting Information). The high‐resolution Fe 2p spectrum of bulk‐Fe_7_Se_8‐x_ (1:8) shows the peaks of Fe^2+^ at 710.7 and 724.2 eV, Fe^3+^ at 712.3 and 726.3 eV, and satellite peaks at 719.2 and 732.1 eV.

Similar XPS features are observed for the other three samples, except for bulk‐Fe_7_Se_8‐x_ (1:4), which shows an additional peak of Fe^0^ at 707.3 eV in the Fe 2p spectrum (Figure 1c).^[^ ^14^ ^]^ For bulk‐Fe_7_Se_8‐x_ (1:8), the Se 3d spectrum (Figure 1d) displays the peaks at 53.5 and 54.4 eV (Se^2−^), 55.3 and 56.4 eV (Se^0^), and a broad peak ≈58.7 eV (SeO_x_).^[^ ^15^ ^]^ The bulk‐Fe_7_Se_8_ (1:4), bulk‐Fe_7_Se_8_ (1:12), and nano‐Fe_7_Se_8_ exhibit similar spectral features. The signal of carbon in nano‐Fe_7_Se_8_ confirms carbon residue from iron acetylacetone (Figure S2, Supporting Information), which reduces the specific capacity. Both XRD and XPS results verify the successful synthesis of all four samples. ICP analysis confirms the Fe/Se atomic ratios of 7:6.97, 7:7.22, 7:7.95 and 7:8.02 for bulk‐Fe_7_Se_8‐x_ (1:4), bulk‐Fe_7_Se_8‐x_ (1:8), bulk‐Fe_7_Se_8_ (1:12), and nano‐Fe_7_Se_8_ (Figure 1e; Table S2, Supporting Information). The Se‐deficiency in bulk‐Fe_7_Se_8‐x_ implies the introduction of Se defects. EPR spectra (Figure 1f) show distinct signals at g = 2.003 for bulk‐Fe_7_Se_8‐x_ (1:4 and 1:8), but not for bulk‐Fe_7_Se_8_ (1:12) or nano‐Fe_7_Se_8_, confirming abundant Se defects in the former two. These defects provide additional sodium storage sites, thereby enhancing the capacities. As presented in Figure 1g–i, bulk‐Fe_7_Se_8‐x_ (1:8) exhibits a hierarchical rugby‐like structure (≈200 µm) composed of irregular particles (≈20 µm). Such a large size originates from the micron‐sized Fe precursor. Notably, macropores are evident between primary particles, and a loose structure is confirmed by Figure S3 (Supporting Information). Elemental mapping shows homogeneous Fe and Se distribution (Figure 1j). Similar micro‐scale morphologies are observed for the bulk‐Fe_7_Se_8‐x_ (1:4) and bulk‐Fe_7_Se_8_ (1:12) (Figure S4, Supporting Information). In contrast, nano‐Fe_7_Se_8_ consists of irregular nanoparticles with an average size of ≈300 nm. HRTEM image (Figure S5b, Supporting Information) reveals distinct lattice fringes of 0.275 and 0.295 nm, corresponding to the (203) and (006) planes. Element mapping confirms homogeneous distribution of Fe, Se, and C elements (Figure S6, Supporting Information). These results verify the successful preparation of bulk and nano Fe_7_Se_8_. TGA (Figure S7, Supporting Information) indicates a high carbon content of 37.8 wt% in nano‐Fe_7_Se_8_, which improves cycling stability but reduces specific capacity.

**Figure 2:**
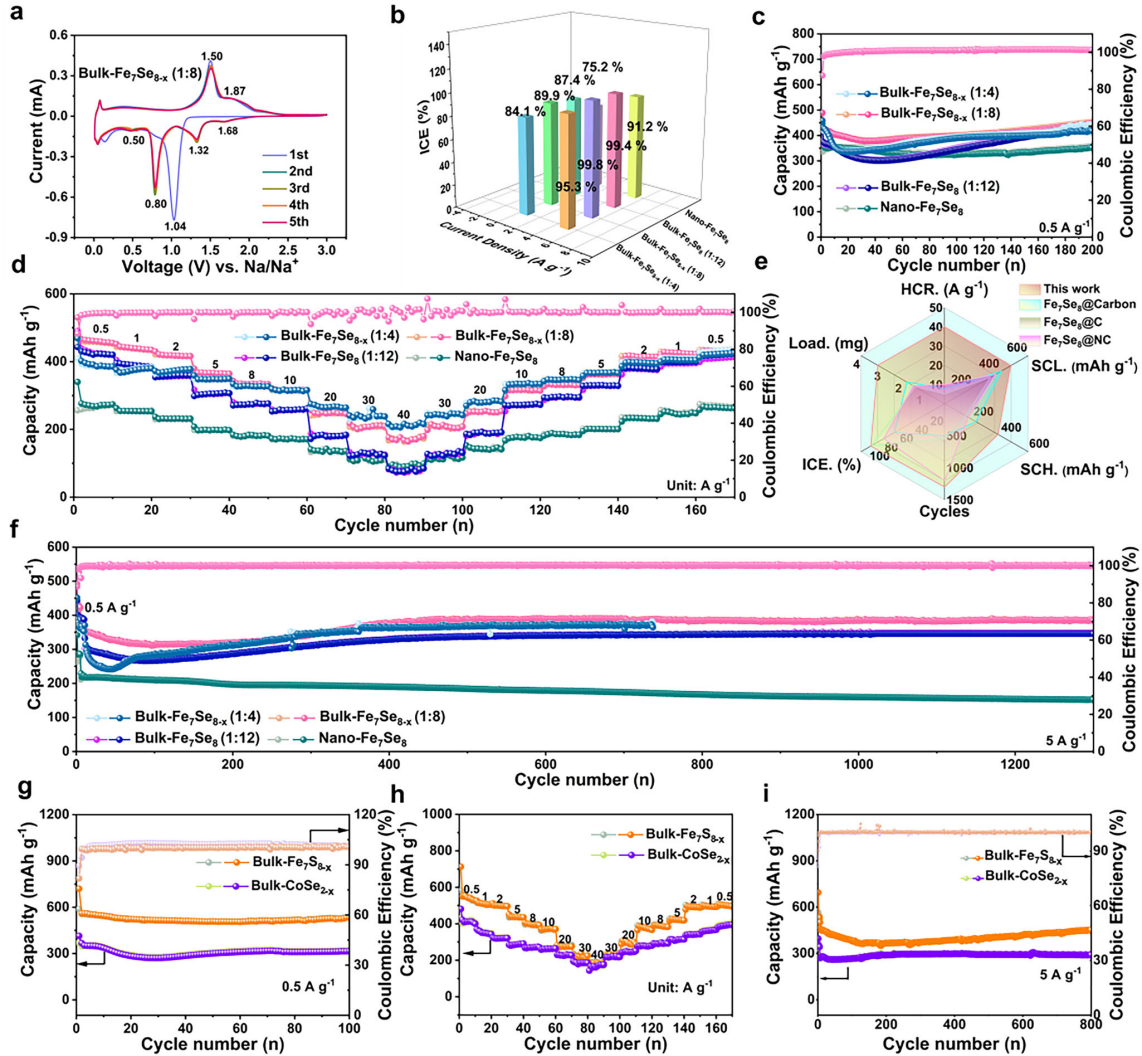
Sodium storage properties investigation. a) CV profiles at 0.1 mV s^−1^ of bulk‐Fe_7_Se_8‐x_ (1:8). b) Initial coulombic efficiencies (ICEs), c) cyclic performances at 0.5 A g^−1^, and d) rate capabilities comparisons. e) Performance comparisons of ICE, highest mass loadings (Load), highest current rate (HCR), specific capacity at 0.5 A g^−1^ (SCL), specific capacity at 5 A g^−1^ (SCH), and cycle numbers (Cycles) with reported Fe_7_Se_8_ based anodes. f) Long‐period cyclic performances comparison at 5.0 A g^−1^. g) Cycle performances at 0.5 A g^−1^, h) rate performances, and i) long‐period cyclic performances at 5.0 A g^−1^ of bulk‐Fe_7_S_8‐x_ and bulk‐CoSe_2‐x_ for SIBs.

To confirm the feasibility of defect‐engineering, the detailed sodium storage performances of Fe_7_Se_8_‐ based anodes were investigated (**Figure** **2**). One obvious reduction peak situated at 1.04 V and a weak peak at ≈0.8 V for the first cathodic scan (Figure 2a) are attributable to the gradual transformation of Fe_7_Se_8_ to Na_2_Se and Fe, as well as the formation of SEI, respectively.^[^ ^16^ ^]^ During the initial anodic scan, one strong peak at 1.50 V and another wide peak situated at 1.87 V are attributed to the gradual oxidation of Na_2_Se and Fe to form FeSe, not Fe_7_S_8_.^[^ ^17^, ^18^, ^19^ ^]^ In the subsequent cathodic scan, the bulk‐Fe_7_Se_8‐x_ (1:8) presents four reduction peaks, the two peaks located at 1.68 V and 1.32 V are possibly resulted from the insertion of Na^+^ into FeSe to form Na_x_FeSe, and the other two peaks at around 0.80 V and 0.50 V are ascribed to the conversion reaction of Na_x_FeSe to form Na_2_Se and Fe.^[^ ^20^ ^]^ Except for the first cycle, the subsequent perfect overlap of CV curves reveals the good reversibility of bulk‐Fe_7_Se_8‐x_ (1:8). With the increase of scan cycles, more redox peaks appear in nano‐Fe_7_Se_8_ (Figure S8a, Supporting Information), implying its more complicated multi‐step redox reaction than bulk‐Fe_7_Se_8‐x_. Specifically, nano‐Fe_7_Se_8_ exhibits three obvious oxidation peaks situated at 1.51, 2.05, and 2.32 V, and five reduction peaks located at 1.95, 1.76, 1.33, 0.80, and 0.60 V. Based on above analysis, the nano‐Fe_7_Se_8_ shows complex reactions, and the superfluous redox peaks at 1.95/2.32 V may correspond to the partial oxidation of Na_2_Se to form Na_2_Se_x_ or Se.^[^ ^13^, ^20^ ^]^ The corresponding voltage platforms originated from charge/discharge curves of two electrodes are consistent with the redox peaks of CV curves (Figures S8b and S9, Supporting Information). The ICE of anode material is an important index for its commercial application. The ICE values of bulk‐Fe_7_Se_8‐x_ (1:4), bulk‐Fe_7_Se_8‐x_ (1:8), bulk‐Fe_7_Se_8_ (1:12) and nano‐Fe_7_Se_8_ is 81.4%, 89.8%, 87.4% and 75.2% at 0.5 A g^−1^, 95.3%, 99.8%, 99.5% and 91.2% at 5 A g^−1^, respectively (Figure 2b). The ICE values of carbon‐free bulk‐Fe_7_Se_8‐x_ (1:8) and bulk‐Fe_7_Se_8_ (1:12) are obviously higher than those of carbon‐rich nano‐Fe_7_Se_8_, hinting the advantage of bulk‐Fe_7_Se_8‐x_. The irreversible capacity loss is possibly originated from the consumption of organic electrolyte to form the SEI film. Additionally, the bulk‐Fe_7_Se_8‐x_ (1:8) manifests a high reversible capacity of 447.3 mAh g^−1^ at 0.5 A g^−1^ after stably cycling for 200 cycles (Figure 2c), superior to those of bulk‐Fe_7_Se_8‐x_ (1:4), bulk‐Fe_7_Se_8_ (1:12), and nano‐Fe_7_Se_8_. The slight capacity attenuation for the first few cycles is possibly a result of the morphology changes due to the huge volume fluctuation, along with the formation of an unstable SEI film. The bulk‐Fe_7_Se_8‐x_ (1:8) also exhibits remarkable rate capability (Figure 2d), the reversible capacities enable it to sustain at 460, 438, 418, 366, 333, and 315 mAh g^−1^ at 0.5, 1, 2, 5, 8, and 10 A g^−1^, respectively. When cycled at the ultra‐high rates of 20, 30, and even 40 A g^−1^, the charge capacity can still retain 248, 207, and 168 mAh g^−1^. Most importantly, as the cyclic rate gradually reduces to 0.5 A g^−1^, the capacity enables recovery to the original level without obvious capacity loss. The rate performance of bulk‐Fe_7_Se_8‐x_ (1:8) is obviously better than that of bulk‐Fe_7_Se_8_ (1:12) and nano‐Fe_7_Se_8_. However, it is noted that although the bulk‐Fe_7_Se_8‐x_ (1:4) manifests lower specific capacities than bulk‐Fe_7_Se_8‐x_ (1:8) at small rates, but its reversible capacities at high rates such as 20, 30 and 40 A g^−1^ are higher than those of bulk‐Fe_7_Se_8‐x_ (1:8). Understandably, the inactive Fe will lower the capacities at low rates, and the richer defects and high‐conductive metallic Fe in bulk‐Fe_7_Se_8‐x_ (1:4) will result in the high‐rate capacities. Moreover, six performance index including ICE, highest mass loading (Load), highest current rate (HCR), specific capacity at a low rate of 0.5 A g^−1^ (SCL), specific capacity at a high rate of 5 A g^−1^ (SCH), as well as cycle numbers (Cycles) are used to measure the application potential of bulk‐Fe_7_Se_8‐x_ (Figure 2e). Compared with reported Fe_7_Se_8_‐based anodes, the bulk‐Fe_7_Se_8‐x_ (1:8) displays the most excellent Na^+^ storage performances.^[^ ^12^, ^21^ ^]^ Besides, Table S3 (Supporting Information) further confirms its outstanding cyclic properties of bulk‐Fe_7_Se_8‐x_, suggesting the positive role of defect‐engineering in promoting sodium storage. Specifically, when the current density is up to 5 A g^−1^ (Figure 2f), the bulk‐Fe_7_Se_8‐x_ (1:8) still maintains a high reversible capacity of 384 mAh g^−1^ even after 1300 long cycles. The capacity difference between bulk‐Fe_7_Se_8_ (1:12) and bulk‐Fe_7_Se_8‐x_ (1:8) is mainly attributable to the rich defects in bulk‐Fe_7_Se_8‐x_ (1:8), which increase the active sites and boost the sodium storage capacity. Whereas nano‐Fe_7_Se_8_ only retains a charge capacity of below 200 mAh g^−1^ under the same testing conditions. A large amount of carbon in nano‐Fe_7_Se_8_ is prone to lowering the charge capacity. The bulk‐Fe_7_Se_8‐x_ (1:4) shows the obvious capacity fluctuation followed by the initial capacity decay and subsequent capacity rise. After 50 cycles, the capacity of bulk‐Fe_7_Se_8‐x_ (1:4) is obviously higher than that of bulk‐Fe_7_Se_8_ (1:12) and nano‐Fe_7_Se_8_. Unluckily, its cycle life is only 730 cycles. Based on the above discussions, the bulk‐Fe_7_Se_8‐x_ (1:8) presents the highest sodium storage capacities and longest high‐rate cyclic life, which results from the cooperative advantages of rich defects and carbon‐free micro‐sized iron selenide. Meanwhile, when the mass loading is as high as 3.32 mg, the bulk‐Fe_7_Se_8‐x_ (1:8) still delivers a high reversible capacity of 446.8 mAh g^−1^ over 200 cycles at 0.5 A g^−1^ (Figure S10, Supporting Information). Furthermore, such a synthesis strategy has been adopted to successfully prepare bulk‐Fe_7_S_8‐x_ and bulk‐CoSe_2‐x_ (Figures S11–S14, Supporting Information). Similar to bulk‐Fe_7_Se_8‐x_, both of them are porous microspheres composed of some primary and irregular particles. Luckily, both bulk‐Fe_7_S_8‐x_ and bulk‐CoSe_2‐x_ exhibit satisfying sodium storage properties (Figure 2g–i; Figures S15 and S16, Supporting Information), both of them exhibit the high ICE values especially at the high rate of 5 A g^−1^ (Figure S15, Supporting Information), stable cyclic performances at both 0.5 and 5 A g^−1^ as well as glorious ultrahigh‐rate capabilities. In comparison with bulk‐CoSe_2‐x_, the bulk‐Fe_7_S_8‐x_ reveals higher reversible capacities than bulk‐CoSe_2‐x_. Its reversible capacity maintains at 533.5 mAh g^−1^ after 100 cycles at 0.5 A g^−1^, and when the current density enhances to 40 A g^−1^, the average capacity enables to retain 180.1 mAh g^−1^ (Figure 2h). Additionally, the bulk‐Fe_7_S_8‐x_ demonstrates a higher sodium‐storage capacity of 450.8 mAh g^−1^ over 800 long cycles at 5 A g^−1^ (Figure 2i). The corresponding high‐loading test at a current density of 0.5 A g^−1^ (Figure S17, Supporting Information) reveals that the bulk‐Fe_7_S_8‐x_ delivers a high charge capacity of 509.3 mAh g^−1^ over 200 cycles. With regard to bulk‐CoSe_2‐x_, despite an initial capacity loss, the reversible capacity is able to stabilize at 370 mAh g^−1^ over 150 cycles. The above remarkable results further disclose the universality of such a synthesis strategy in this work.

**Figure 3:**
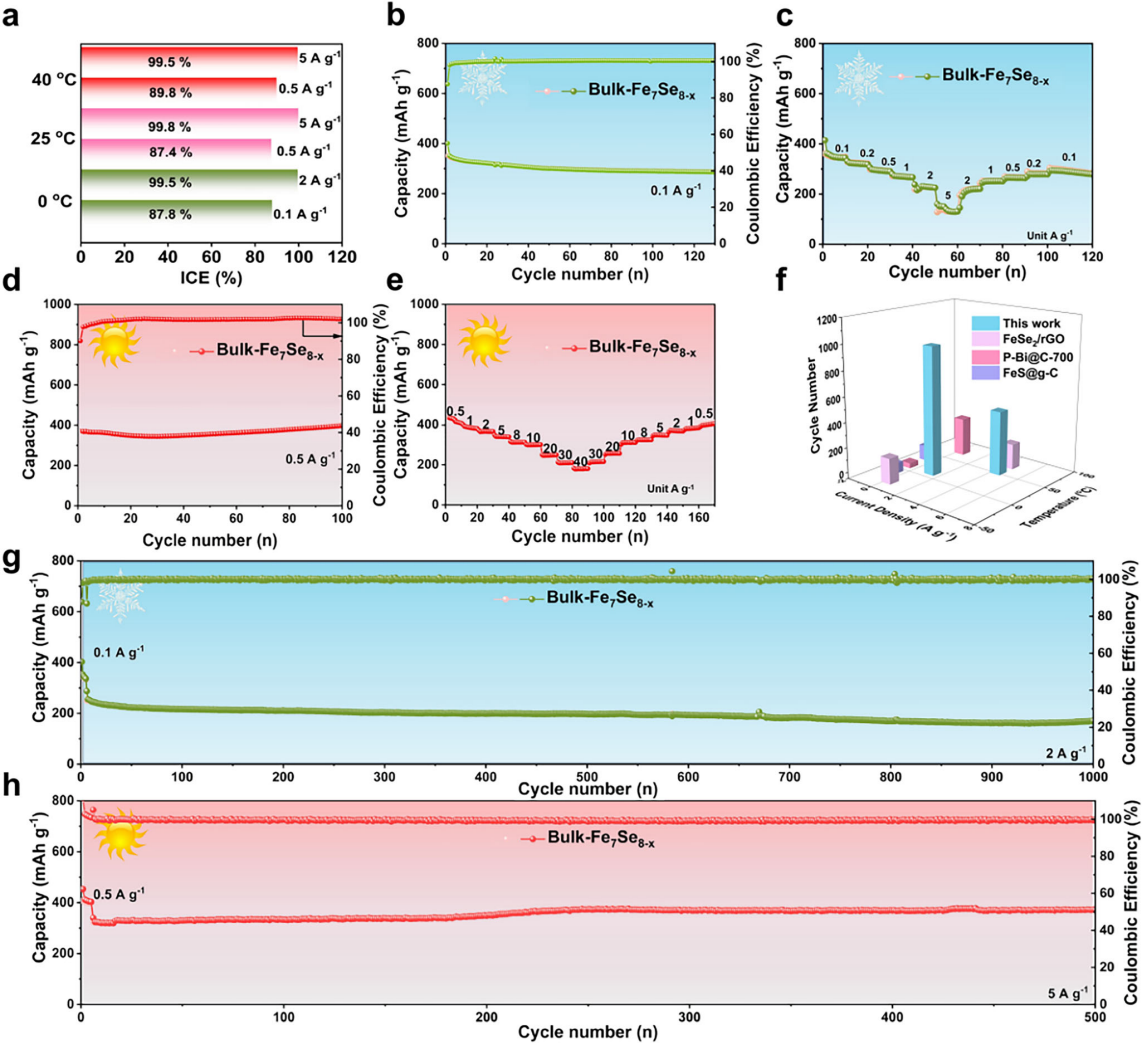
High/low‐temperature properties of bulk‐Fe_7_Se_8‐x_ (1:8). a) ICEs at different test temperatures, b, c) cyclic performance at 0.1 A g^−1^ and rate capability at 0 °C, d, e) cyclic performance at 0.5 A g^−1^ and rate capability at 40 °C, f) performance comparison with reported wide‐temperature anode materials, g, h) high‐rate cyclic performances at 0 and 40 °C.

The high/low‐temperature sodium ion storage is another important indicator to verify the practicability of anode material for SIBs. The sodium storage properties of bulk‐Fe_7_Se_8‐x_ (1:8) at 0 and 40 °C were investigated in detail to evaluate its temperature adaptability (**Figure** **3**; Figure S18, Supporting Information). According to Figure 3a, the bulk‐Fe_7_Se_8‐x_ (1:8) shows high ICEs at three test temperatures. The high‐rate ICE can reach as high as 99.8% at 25 °C. As shown in Figure 3b, when cycled at 0 °C, the bulk‐Fe_7_Se_8‐x_ (1:8) reveals a satisfying specific capacity of 286 mAh g^−1^ for 130 cycles at 0.1 A g^−1^. When cycled at 40 °C, a higher reversible capacity of 396.8 mAh g^−1^ is retained over 100 cycles at 0.5 A g^−1^ for bulk‐Fe_7_Se_8‐x_ (1:8) (Figure 3d). Additionally, bulk‐Fe_7_Se_8‐x_ (1:8) also exhibits excellent rate properties at both extreme temperatures (Figure 3c,e). Compared with the reported anodes (Figure 3f), the bulk‐Fe_7_Se_8‐x_ (1:8) displays the most excellent high/low‐temperature performances.^[^ ^22^, ^23^, ^24^ ^]^ Surprisingly, the bulk‐Fe_7_Se_8‐x_ (1:8) still demonstrates excellent high‐rate cyclic stability with a long life‐span (170.6 mAh g^−1^ over 1000 cycles at 2 A g^−1^, Figure 3g) at 0 °C. More impressively, the bulk‐Fe_7_Se_8‐x_ (1:8) demonstrates a high reversible capacity of 371.7 mAh g^−1^ at 5 A g^−1^ over 500 cycles at 40 °C, comparable to that of 25 °C (Figure 3h). Such excellent temperature adaptability further manifests the potential applications of bulk‐Fe_7_Se_8‐x_ in SIBs.

**Figure 4:**
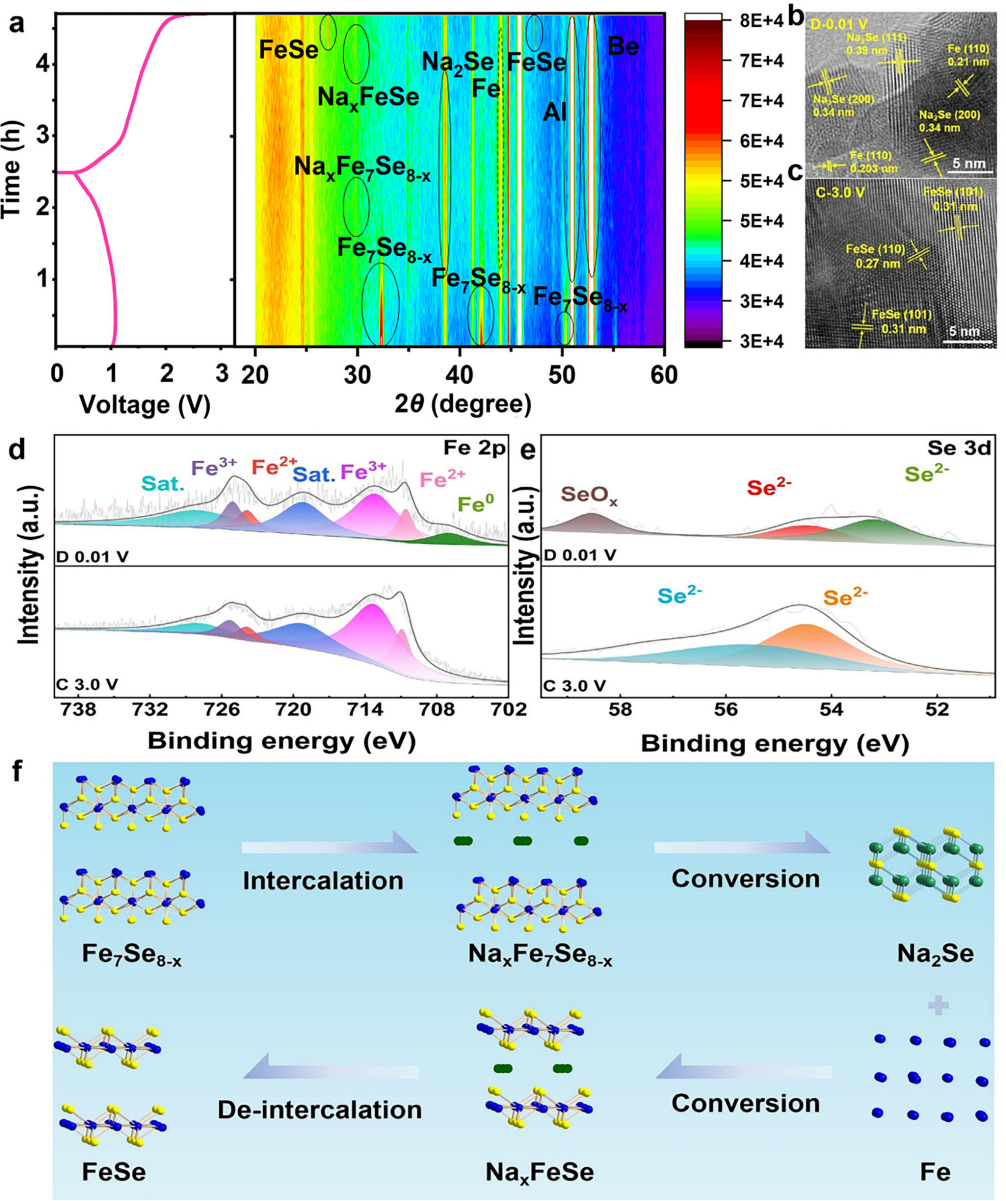
Reaction mechanism of bulk‐Fe_7_Se_8‐x_. a) In situ XRD patterns, b, c) *ex situ* HRTEM images, d, e) *ex situ* XPS spectra of Fe 2p and Se 3d, and f) diagram of reaction mechanism.

Such remarkable sodium storage properties of bulk‐Fe_7_Se_8‐x_ inspire us to explore its reaction mechanism. Herein, some in situ XRD, *ex situ* HRTEM, and XPS techniques were employed to elucidate the electrochemical reaction of bulk‐Fe_7_Se_8‐x_. The in situ XRD patterns in **Figure** **4**a reveal that when the original bulk‐Fe_7_Se_8‐x_ (1:8) electrode discharges to 1.04 V, the Na^+^ intercalation reaction first occurs to form Na_x_Fe_7_Se_8‐x_, and after discharging to 0.01 V, the conversion reaction occurs to form the final Na_2_Se and metallic Fe (Figure S19, Supporting Information). When the bulk‐Fe_7_Se_8‐x_ (1:8) is charged to 1.5 V, except for the existence of Na_2_Se and metallic Fe, the Na_x_FeSe also appears.^[^ ^11^, ^25^ ^]^ Moreover, when charging to 3.0 V, two new diffraction peaks are attributable to the newly formed FeSe. The HRTEM images were further employed to verify the phases. When discharging to 0.01 V (Figure 4b,c), the clear crystal lattices with the interlayer spacings of 0.34 and 0.39 nm correspond to the (220) and (111) planes of Na_2_Se, another interlayer spacing of 0.21 nm belongs to the (110) plane of metallic Fe. The fully charged bulk‐Fe_7_Se_8‐x_ (1:8) electrode exhibits two clear crystal lattices with the interlayer spacings of 0.31 and 0.27 nm, corresponding to the (101) and (110) planes of FeSe.^[^ ^26^, ^27^, ^28^ ^]^ Moreover, the reaction mechanism of bulk‐Fe_7_Se_8‐x_ was also studied via *ex situ* XPS. When the bulk‐Fe_7_Se_8‐x_ (1:8) is fully discharged to 0.01 V (Figure 4d,e), the Fe 2p spectrum shows seven fitted peaks, aside from the six peaks of Fe^2+^, Fe^3+,^ along with two satellite peaks, a new peak situated at 707 eV is ascribed to the metallic Fe^0^. When fully charging to 3.0 V, the metallic Fe^0^ vanishes.^[^ ^13^, ^14^ ^]^ The Se 3d spectrum consists of two sharp peaks located at 54.5 and 55.3 eV for the deep discharge progress, which are attributable to Na_2_Se.^[^ ^29^, ^30^, ^31^ ^]^ In contrast with the discharged state, two peaks for the charged bulk‐Fe_7_Se_8‐x_ (1:8) move to the high binding energies, hinting at the formation of FeSe (Figure 4e),^[^ ^9^, ^17^, ^19^ ^]^ which is mainly resulted from the more stable FeSe and lower formation energy of FeSe reacted from Fe and Na_2_Se. Both thermodynamic and kinetic factors lead to the phase change from Fe_7_Se_8‐x_ to FeSe.^[^ ^18^, ^32^, ^33^ ^]^ Moreover, the reversible reaction mechanism of bulk‐Fe_7_Se_8‐x_ (1:8) was further verified from the derivative plots (dQ/dV) and discharge/charge profiles (Figure S20, Supporting Information). The overlap of redox peaks in the CV curves from the 2nd cycle to 100th cycle reveals its high reversibility.^[^ ^33^ ^]^ The detailed reaction equations of bulk‐Fe_7_Se_8‐x_ are shown in Figure 4f.^[^ ^9^, ^22^ ^]^

**Figure 5:**
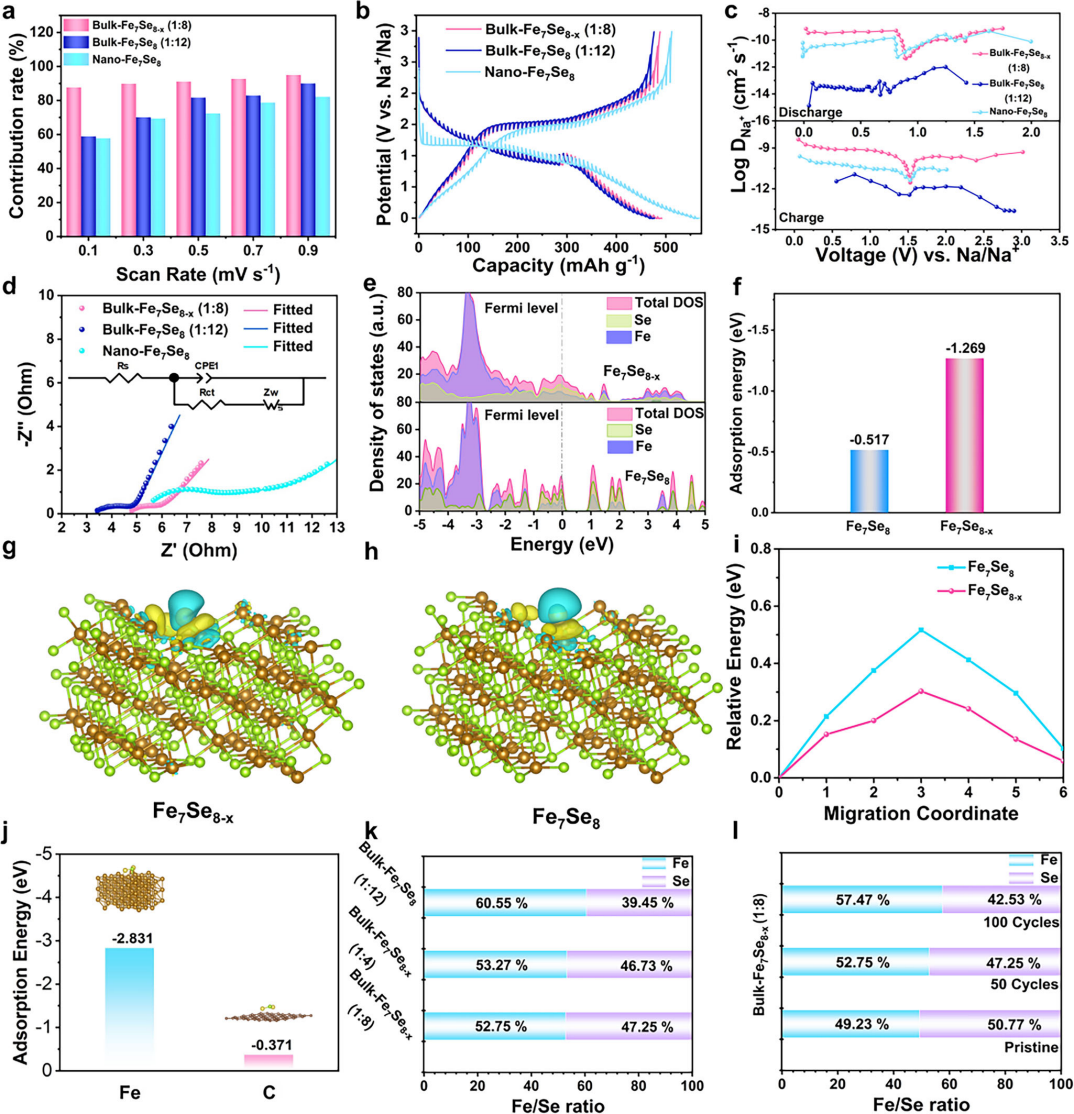
Kinetics analysis of bulk‐Fe_7_Se_8‐x_. a) Capacitance contribution ratios, b) GITT profiles, and c) Na‐ion diffusion coefficients of discharge/charge process, d) Nyquist plots at the 100th cycle of three electrodes, e) density of states (DOS), f) absorption energies, g, h) charge density differences, and i) Na^+^ migration energy barriers of defect‐rich Fe_7_Se_8‐x_ and defect‐free Fe_7_Se_8_, j) adsorption energies of Na_2_Se by Fe and C, k) atomic ratios of Fe/Se for three electrodes after 50 cycles, l) atomic ratios of Fe/Se for bulk‐Fe_7_Se_8‐x_ (1:8) after different cycles.

The electrochemical kinetics of bulk‐Fe_7_Se_8‐x_ (1:8) was deeply discussed to validate its remarkable sodium storage properties. First, the CV curves at various scan rates (from 0.1 to 0.9 mV s^−1^) were utilized to analyze the charge storage mechanism (Figure S21a, Supporting Information). The calculated b values of four peaks for bulk‐Fe_7_Se_8‐x_ (1:8) are 0.70, 0.59, 0.82, and 0.96, respectively (Figure S21b, Supporting Information). It can be concluded that both diffusion behavior and pseudocapacitance behavior contribute to the charge storage process. According to **Figure** **5**a and Table S4, with the increase of scan rate, the capacitance contributions of all three electrodes gradually enhance. By comparison, we find that the pseudocapacitance contribution of bulk‐Fe_7_Se_8‐x_ (1:8) is distinctly larger than those of bulk‐Fe_7_Se_8_ (1:12) and nano‐Fe_7_Se_8_ at the same scan rate. The capacitance value of bulk‐Fe_7_Se_8‐x_ (1:8) is as high as 95.0% at 0.9 mV s^−1^, revealing that the capacitance plays a major role in the whole electrochemical storage process. As observed from the CV curve of bulk‐Fe_7_Se_8‐x_ (1:8) at 0.7 mV s^−1^ (Figure S21c, Supporting Information), the rosy part originates from the capacitance contribution; the high ratio of 93.7% indicates that capacitance dominates the entire charge storage process of the bulk‐Fe_7_Se_8‐x_ (1:8) electrode. Whereas the bulk‐Fe_7_Se_8_ (1:12) and nano‐Fe_7_Se_8_ show lower capacitance ratios of 82.9% and 78.7% (Figures S22c and S23c, Supporting Information), which further explains the reason why the bulk‐Fe_7_Se_8‐x_ owns a distinctly higher capacity and excellent rate capability than the other two controls.^[^ ^34^, ^35^, ^36^, ^37^ ^]^ Anyway, the other kinetic factors will also affect the sodium storage properties. Furthermore, the GITT tests were employed to investigate the Na^+^ diffraction kinetics (Figure 5b). GITT curves reveal that both bulk‐Fe_7_Se_8‐x_ (1:8) and bulk‐Fe_7_Se_8_ (1:12) deliver smaller over‐potentials than nano‐Fe_7_Se_8_ for both discharge and charge processes, indicating their fast Na^+^ diffusion kinetics. Based on the calculated Na^+^ diffusion coefficients (Figure 5c), the bulk‐Fe_7_Se_8‐x_ (1:8) presents better Na^+^ diffusion kinetics than bulk‐Fe_7_Se_8_ (1:12) and nano‐Fe_7_Se_8_ for both discharge and charge processes. Moreover, the Nyquist plots contain a semicircle in the high‐frequency region and an inclined line in the low‐frequency region, where the depressed semicircles are ascribed to the charge transfer impedance, and the sloping lines are derived from the Na^+^ diffusion process.^[^ ^38^ ^]^ The EIS data reveal that all three electrodes deliver a low charge transfer impedance after 100 cycles, indicating that the Fe_7_Se_8_ with an inherent high conductivity can verify the highly efficient electron transfer under the assistance of ether‐like electrolyte. In comparison with bulk‐Fe_7_Se_8_ (1:12) and nano‐Fe_7_Se_8_, the defective bulk‐Fe_7_Se_8‐x_ (1:8) manifests a smaller R_ct_ value and a lower slope (Figure 5d; Table S5, Supporting Information), demonstrating its rapid electron transfer and Na^+^ diffusion kinetics due to the introduction of Se defects.

Beyond the above kinetics investigations, the DFT calculations further corroborate the exceptional sodium storage capabilities of bulk‐Fe_7_Se_8‐x_. Notably, the Se‐defect‐rich Fe_7_Se_8‐x_ reveals a higher conductivity than defect‐free Fe_7_Se_8_ at the Fermi energy level (Figure 5e).^[^ ^19^, ^20^ ^]^ According to Figure 5f, the adsorption energy of defect‐rich Fe_7_Se_8‐x_ is obviously higher than that of defect‐free Fe_7_Se_8_, indicating that Fe_7_Se_8‐x_ is easier to adsorb Na^+^ to provide higher sodium storage capacities, in accordance with experimental results. The differential charge density images reveal that Fe_7_Se_8‐x_ benefits the charge transfer due to the introduction of defects (Figure 5g,h). Furthermore, the Fe_7_Se_8‐x_ exhibits a smaller sodium migration energy barrier than Fe_7_Se_8_ (Figure 5i), suggesting the rapider Na^+^ diffusion kinetics due to the presence of Se defects in the Fe_7_Se_8_ crystal structure (Figure S24, Supporting Information). The above theoretical results commonly explain the reason for the superior sodium storage capabilities of Fe_7_Se_8‐x_.^[^ ^39^ ^]^ To identify the key roles of Fe and C in sodium storage properties, the corresponding adsorption energies of Na_2_Se by Fe and C were calculated (Figure 5j). Notably, the adsorption energy of Fe on Na_2_Se is −2.831 eV, obviously higher than that of C (−0.371 eV), suggesting the positive role of extra Fe in promoting sodium storage properties. After 50 cycles, bulk‐Fe_7_Se_8‐x_ (1:4), bulk‐Fe_7_Se_8‐x_ (1:8), and bulk‐Fe_7_Se_8_ (1:12) demonstrate different Se losses (Figure 5k; Tables S2 and S6, Supporting Information). The bulk‐Fe_7_Se_8‐x_ (1:12) presents the highest Se loss, and the bulk‐Fe_7_Se_8‐x_ (1:4) shows the lowest Se loss due to the presence of extra Fe, which enables anchoring polyselenide and decreases Se loss. As for bulk‐Fe_7_Se_8‐x_ (1:8), with the increase of cycle numbers, the speed of Se loss obviously decreases (Figure 5l). The Se loss will lead to the capacity decay and meanwhile provide extra Fe to gradually restrain the dissolution of polyselenide, and thus, achieve subsequent stable cyclic performance. Anyway, the uniform distributions of Fe and Se after cycling confirm the good stability of above electrodes (Figure S25, Supporting Information). Both experimental results and theoretical calculations indicate the positive role of metallic Fe in promoting sodium storage properties.

Such excellent performances of bulk‐Fe_7_Se_8‐x_ were further verified via the interface composition and morphology evolution. As observed from HRTEM images (Figure S26, Supporting Information), the surface SEI layer of bulk‐Fe_7_Se_8‐x_ (1:8) is thin and uniform (around 10 nm), which is more stable and enables to guaranteeing the fast Na^+^ transmission and good structural stability, therefore exhibiting excellent rate performance and excellent cyclic stability.^[^ ^40^, ^41^ ^]^ Moreover, *ex‐situ* XPS spectra confirm the composition of the SEI film (Figure S27, Supporting Information). When deeply discharging to 0.01 V, the Na 1s spectrum reveals that abundant inorganic components such as NaF and Na_2_CO_3_ are formed on the surface of bulk‐Fe_7_Se_8‐x_ (1:8), dependent on the ether‐like electrolyte.^[^ ^42^, ^43^ ^]^ According to the C 1s spectrum (Figure S27b, Supporting Information), five peaks corresponding to the Na_x_‐HC, C─C/C─H, C─O, C═O, and ─CO_3_ are observed for the discharged state. When charged to 3 V, the content of Na_2_CO_3_ will obviously decrease, indicating that some components of the SEI layer will partly dissolve.^[^ ^44^, ^45^, ^46^ ^]^ The SEM cross‐section images of fresh and cycled bulk‐Fe_7_Se_8‐x_ (1:8) electrode (Figure S28, Supporting Information) show that the cross‐section is very flat and the contact part with Cu foil is tight, and the thickness of cycled bulk‐Fe_7_Se_8_ (1:8) is ≈55.4 µm, which is slightly thicker than that of fresh one due to the volume changes.^[^ ^47^ ^]^ The surface SEM images of bulk‐Fe_7_Se_8‐x_ (1:8) after different cycles are depicted in Figure S29 (Supporting Information). It is clearly noted that the shape and outline of bulk‐Fe_7_Se_8‐x_ (1:8) are basically retained after one cycle (Figure S29a, Supporting Information). After 100 cycles, the micro‐sized structure will gradually change into even and small nanoparticles (Figure S29b, Supporting Information).^[^ ^48^ ^]^ Based on the above analysis, the defective bulk‐Fe_7_Se_8‐x_ not only enables to construction of a stable and thin SEI film to fasten reaction kinetics, but also the formed even‐dispersed nanoparticles can suppress the structural pulverization/collapse, and maintain the stable electrode structure. All these factors further explain why the bulk‐Fe_7_Se_8‐x_ (1:8) owns such excellent electrochemical performances for SIBs.

The above viewpoints can be used to explain the capacity fluctuation of bulk Fe_7_Se_8‐x_ or bulk‐Fe_7_Se_8_. The bigger capacity fluctuation for bulk Fe_7_Se_8‐x_ (1:4) in comparison with the other counterparts is attributable to the relatively unstable electrode structure due to the introduction of higher Se defects. As the conversion‐based anode, the huge volume changes upon charging/discharging cycles will greatly induce the structural degradation and material pulverization, ultimately resulting in the obvious capacity fading. After a few cycles, the micron‐sized bulk‐Fe_7_Se_8‐x_ (1:4) will break down into numerous nanoparticles. Meanwhile, due to the slow dissolution of Se‐based species, the superfluous Fe will uniformly mix with newly formed Na_2_Se, which enables anchoring Se to suppress the dissolution of polyselenides.^[^ ^49^, ^50^, ^51^, ^52^ ^]^ Additionally, the pseudocapacitive contributions obviously enhance due to the so‐called “grinding effect” upon repeated cycles, finally contributing to the capacity rise.

**Figure 6:**
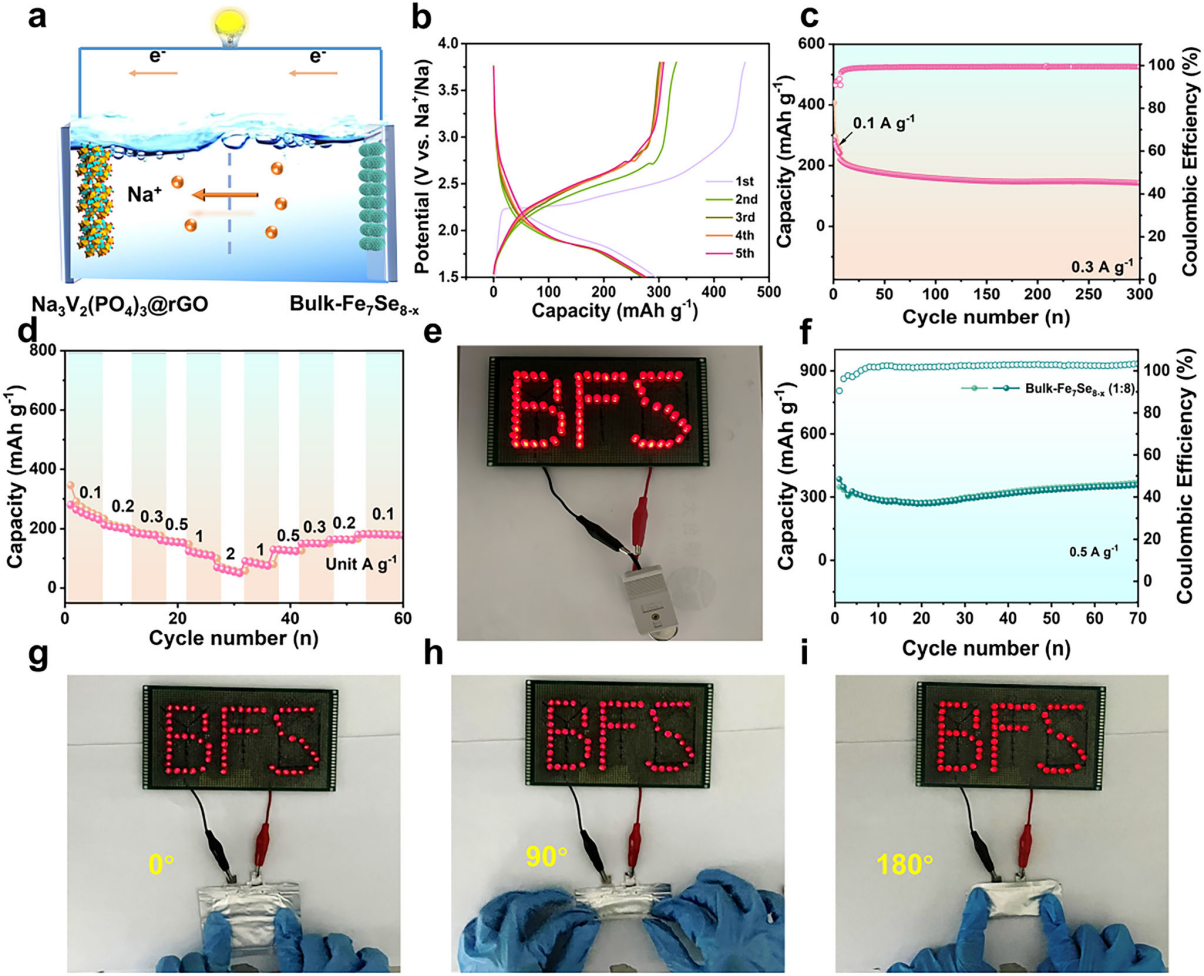
Electrochemical properties of Na_3_V_2_(PO_4_)_3_@rGO//bulk‐Fe_7_Se_8‐x_ full battery, a) schematic diagram of full battery, b) discharge/charge profiles at 0.1 A g^−1^, c) cycling performance at 0.3 A g^−1^, d) rate capability, e) LEDs powered by sodium‐ion button full battery, f) cyclic performance of bulk‐Fe_7_Se_8‐x_ (1:8) based pouch battery at 0.5 A g^−1^, g–i) photos of LEDs powered by pouch battery being bent to 0°, 90°, and 180°.

To further study the potential commercial application of bulk‐Fe_7_Se_8‐x_, the Na^+^ full battery (Na_3_V_2_(PO_4_)_3_@rGO//bulk‐Fe_7_Se_8‐x_, **Figure** **6**a) was assembled by matching bulk‐Fe_7_Se_8‐x_ (1:8) anode with Na_3_V_2_(PO_4_)_3_@rGO cathode (Figure S30a–d, Supporting Information). In our case, the test voltage range is 1.5–3.8 V. Figure 6b reveals that the initial discharge capacity is close to 300 mAh g^−1^ at 0.1 A g^−1^. The charge and discharge platforms are obvious in the suitable voltage range under the assistance of an ether‐like electrolyte. The discharge capacity reaches as high as 254.9 mAh g^−1^ at 0.1 A g^−1^ for 50 cycles (Figure S31, Supporting Information). Besides, the full battery shows long‐term cyclic stability at 0.3 A g^−1^, and the discharge capacity can remain at 142.5 mAh g^−1^ over 300 cycles (Figure 6c). The rate performance of the full battery presents that the reversible capacity can reach 284.6, 251, 220.9, 165.4, 65.1, and 25.1 mAh g^−1^ at the current rates of 0.1, 0.2, 0.3, 0.5, 1, and even 2 A g^−1^. When the current density gradually restores to the original 0.1 A g^−1^, no obvious capacity loss is observed, revealing its good reversibility (Figure 6d). Surprisingly, our assembled Na_3_V_2_(PO_4_)_3_@rGO//bulk‐Fe_7_Se_8‐x_ button full battery can easily power the “BFS” logo assembled by 65 small light‐emitting‐diode (LED) bulbs (Figure 6e), manifesting the potential for various portable devices. Additionally, we assembled a flexible sodium ion battery with bulk‐Fe_7_Se_8‐x_ as the anode material. As shown in Figure 6f, the soft‐package sodium‐ion battery enables to delivery of a high capacity of 355 mAh g^−1^ over 70 cycles at 0.5 A g^−1^. Moreover, to confirm its mechanical stability, we bent the flexible battery at different angles. The curving flexible battery can still easily illuminate the LED bulbs (Figure 6g–i) even being bent to 90° and 180°, which further demonstrates its potential in the field of flexible sodium‐ion batteries.^[^ ^53^, ^54^ ^]^

## 3 Conclusion

In summary, under the guidance of defect engineering, the bulk‐Fe_7_Se_8‐x_ with controllable defects and carbon‐free design has been rationally synthesized via a one‐step calcination reaction. As promising anode materials for SIBs, the optimized bulk‐Fe_7_Se_8‐x_ realizes outstanding fast‐charging ability and good temperature adaptability. Specifically, it delivers higher reversible capacity (460 mAh g^−1^/0.5 A g^−1^ at 25 °C), more remarkable cyclic stability (384 mAh g^−1^ after 1300 cycles at 5 A g^−1^ and 25 °C, 170.6 mAh g^−1^ after 1000 cycles at 2 A g^−1^ and 0 °C, and 371.7 mAh g^−1^ after 500 cycles at 5 A g^−1^ and 40 °C), and more excellent rate performance (168 mAh g^−1^ at 40 A g^−1^ and 25 °C) than bulk‐Fe_7_Se_8_ and carbon‐rich nano‐Fe_7_Se_8_. Furthermore, in‐situ XRD and *ex‐situ* HRTEM/XPS techniques indicate that the bulk‐Fe_7_Se_8‐x_ transforms into a new phase of FeSe after the first charge process. The *ex‐situ* HRTEM images and SEM cross‐section/surface images demonstrate that bulk‐Fe_7_Se_8‐x_ is able to form a stable and thin SEI layer on the electrode interface, and maintain a stable electrode structure. Meanwhile, various kinetic strategies coupled with DFT calculations deeply confirm the rapid Na^+^ diffusion/reaction kinetics and high‐efficiency electronic transfer process of bulk‐Fe_7_Se_8‐x_. Moreover, the origin of remarkable sodium storage properties for bulk‐Fe_7_Se_8‐x_ has been elucidated. The above universal and low‐cost synthesis strategy has been extended to prepare the fast‐charging defective bulk‐Fe_7_S_8‐x_ and bulk‐CoSe_2‐x_ anodes. Additionally, the assembled high‐performance button full batteries and flexible SIBs enable powering the “BFS” LED logos. This work provides an effective method for the low‐cost preparation of high‐performance conversion‐type electrodes in energy storage fields.

## 4 Experimental Section

### Synthesis of Bulk‐Fe_7_Se_8‐x_

Typically, the reduced iron powder (CP, 100 mesh, Shanghai Aladdin Bio‐Chem Technology Co. Ltd.) and selenium powder (*≥*99.9%, Shanghai Macklin Biochemical Co., Ltd.) with a weight ratio of 1:8 or 1:4 were physically mixed for several minutes in a mortar, then the obtained mixture was placed in a porcelain boat covered with tin foil to verify the sufficient reaction. After that, the mixture was kept at 650 °C for 10 h under the protection of an Ar atmosphere with a heating rate of 10 °C min^−1^. Later, the tin foil was removed, and the following step was conducted to remove the excess selenium powder to obtain bulk‐Fe_7_Se_8‐x_. The bulk‐Fe_7_Se_8_ was synthesized via a similar preparation process except for changing the Fe/Se ratio to 1:12.

### Synthesis of Nano‐Fe_7_Se_8_

The nano‐Fe_7_Se_8_ was also prepared as the contrast sample. Typically, the iron acetylacetone (*≥*98%, Shanghai Macklin Biochemical Co., Ltd.) and selenium powder (*≥*99.9%, Shanghai Macklin Biochemical Co., Ltd.) with a weight ratio of 1:2 were physically mixed in a mortar. After that, the mixture was placed in a porcelain boat and kept at 500 °C for 4 h in an Ar/H_2_ atmosphere to obtain nano‐Fe_7_Se_8_.

### Synthesis of Bulk‐Fe_7_S_8‐x_

The preparation method is similar to bulk‐Fe_7_Se_8‐x_; the only difference is that selenium powder was replaced by sublimed sulfur (CP, Sinopharm Chemical Co. Ltd.) during the reaction.

### Synthesis of Bulk‐CoSe_2‐x_

Typically, the reduced cobalt powder (*≥*99.5%, Shanghai Macklin Biochemical Co., Ltd.) and selenium powder (*≥*99.9%, Shanghai Macklin Biochemical Co., Ltd.) with a weight ratio of 1:8 were physically mixed for several minutes in a mortar, then the obtained mixture was placed in a porcelain boat covered with tin foil to verify the sufficient reaction. After that, the mixture was kept at 400 °C for 10 h under the protection of an Ar atmosphere with a heating rate of 10 °C min^−1^. Later, the tin foil was removed, and the following step was conducted to remove excess selenium powder to obtain bulk‐CoSe_2‐x_.

### Materials Characterization

The crystal structures of the as‐prepared products were characterized by X‐ray diffraction (Bruker D8 Advance, USA). The morphologies of all the samples were observed with the FESEM (JSM‐7610F, Japan) equipped with an energy dispersive spectroscopy (EDS) detector. JEOL‐1400Plus transmission electron microscope (TEM, 120 kV, Japan) and high‐resolution TEM (HRTEM, JEM‐2100F, 200 kV, Japan) were also used to observe the microstructures and analyze the structural changes. The chemical states of all elements were recorded by the X‐ray photoelectron spectroscopy (XPS, Thermo Scientific, ESCALAB X^+^, USA). The atomic ratio of Fe/Se was verified via the inductively coupled plasma emission spectrometer (ICPAES, iCAP PRO, American Thermo Fisher). The carbon contents in three samples were confirmed by a thermal analyzer (TGA, Netzsch Sta 449F3, Germany). The defects were analyzed by electron paramagnetic resonance (EPR) spectroscopy (JES‐FA2000, JEOL, Japan).

## Conflict of Interest

The authors declare no conflict of interest.

## Supporting information



Supporting Information

## Data Availability

The data that support the findings of this study are available from the corresponding author upon reasonable request.
