# Supplementary material for: Defect‐Engineered Bulk Conversion Anodes for Fast and Temperature‐Adaptive Na+ Storage
Source: Adv Sci (Weinh). 2025 Oct 17;13(1):e15276. doi: 10.1002/advs.202515276 (PMC12767120; doi:10.1002/advs.202515276)
Supplement: Supplementary file 1 — Supporting Information [file ADVS-13-e15276-s001.docx]

**Supporting Information**

**Defect-Engineered Bulk Conversion Anodes for Fast and Temperature-Adaptive Na^+^ Storage**

Yanli Zhou^*,+^, Ao Xu^+^, Zhiqi Li, Yifei Wang, Jiawen Yan, Fuyi Jiang, Wei Liu, Xin Gu^*^, Huan Pang, and Jian Yang^*^

Y. Zhou, A. Xu, Z. Li, Y. Wang, J. Yan, F. Jiang, W. Liu
Shandong Key Laboratory of Advanced Structural Materials Genome Engineering, School of Environmental and Material Engineering, Yantai University, Yantai 264005, Shandong, China
E-mail: [zhouyanli@ytu.edu.cn](mailto:zhouyanli@ytu.edu.cn) (Y. Zhou)

J. Yang
Key Laboratory of Colloid and Interface Chemistry, Ministry of Education, School of Chemistry and Chemical Engineering, Shandong University, Jinan 250100, China
E-mail: [yangjian@sdu.edu.cn](mailto:yangjian@sdu.edu.cn) (J. Yang)

X. Gu

Shandong Key Laboratory of Advanced Electrochemical Energy Storage Technologies, College of New Energy, State Key Laboratory of Heavy Oil Processing, China University of Petroleum (East China), Qingdao 266580, Shandong, China

1. mail: [guxin@upc.edu.cn](mailto:guxin@upc.edu.cn) (X. Gu)

H. Pang

School of Chemistry and Chemical Engineering, Yangzhou University, Yangzhou 225009, Jiangsu, China

^+^ These authors contributed equally to this work.

**Part I:**

**1.1. Electrochemical measurements**

The working electrode was made by mixing active materials, acetylene black, and sodium carboxymethycellulose (CMC) with a weight ratio of 8:1:1 in the deionized water, and the obtained sticky paste was uniformly coated on the clean Cu foil and dried in air for 6 h, then cut into the disc with a diameter of 12 mm. Except for the high mass loading, the average mass loading of active materials was around 1.0-1.2 mg cm^-2^. To perform the electrochemical measurements for SIBs, the CR2032-type Na-ion coin cells were assembled by using glass fiber (Whatman, GF/F) as a separator, a fresh metal sodium sheet with a diameter of 14 mm was used as the counter electrode, and 1 M NaPF_6_ in diethylene glycol dimethyl ether (DIGLYME) as the electrolyte. The full cells were assembled by selecting the Na_3_V_2_(PO_4_)_3_@rGO as the cathode to match with the bulk-Fe_7_Se_8-x_ anode.^[1]^ The galvanostatic charge/discharge tests were performed with the Land CT2001A testing system. The cyclic voltammetry (CV) tests and electrochemical impedance spectra (EIS) were carried out using the CHI 660E and AUTOLABPGSTAT302N electrochemical workstations. All the electrochemical measurements were carried out at 25 °C (room temperature), 0 °C (low/cold temperature) and 40 ^o^C (high temperature).

**1.2. Kinetics analysis**

Formula (1) gives the relationship between current (i) and scan rate (v).^[2, 3]^

$\text{i}\text{=}\text{a}\text{v}^{\text{b}}$ (1)

The b value is determined by the plot of log i vs. log v. Where a and b are fitting coefficients. b=0.5 implies the Na^+^ extraction/insertion process is controlled by the diffusion process. Whereas the b value of 1.0 represents the pseudocapacitance controlled process.

The following equation (2) is employed to further calculate and investigate the pseudocapacitance contribution of overall capacity.^[4]^

$\text{i}\text{=}\text{k}_{\text{1}}\text{v}\text{+}\text{k}_{\text{2}}\text{v}^{\frac{\text{1}}{\text{2}}}$ (2)

In this equation,$\text{ }\text{k}_{\text{1}}\text{v}$represents the capacity contribution from pseudo-capacitance behavior, and $\text{k}_{\text{2}}\text{v}^{\text{1/2}}$ expresses the capacity contribution from diffusion process.

According to the Fick's second law, when voltage is proportional to τ^1/2^, D_Na_^+^ can be calculated according to the following formula (3):^[5]^

$\begin{aligned} \text{D}_{\text{Na}^{\text{+}}}\text{=}\frac{\text{4}}{\text{πτ}}\left( \frac{\text{m}_{\text{B}}\text{V}_{\text{M}}}{\text{M}_{\text{B}}\text{S}} \right)^{\text{2}}\left( \frac{{\text{∆}\text{E}}_{\text{S}}}{{\text{∆}\text{E}}_{\text{t}}} \right)^{\text{2}}\text{ }\left( \text{3} \right) \end{aligned}$

**1.3. Computational details**

All DFT calculations were performed using the Vienna Ab initio Simulation Package (VASP). The projector augmented wave (PAW) pseudopotential with the PBE generalized gradient approximation (GGA) exchange correlation function was utilized in the computations.^[6, 7]^ The cutoff energy of plane waves basis set was 520 eV and a Monkhorst-Pack mesh of 3×3×1 was used in K-sampling. All structures were spin polarized and all atoms were fully relaxed with the energy convergence tolerance of 10^-5^ eV per atom, and the final force on each atom was < 0.05 eV Å^-1^. Finally, the adsorption energies (E_ads_) were calculated as E_ads_= E_ad/sub_ -E_ad_ -E_sub_, where E_ad_/_sub_, E_ad_, and E_sub_ were the total energies of the optimized adsorbate/substrate system, the adsorbate in the structure, and the clean substrate, respectively. The Climbing Image-Nudged Elastic Band methods have been employed to calculate the Na ions migration barriers in the structures.^[8]^

**Part II: Supplementary Results**

**Table S1.** Structural parameters of Fe_7_Se_8_.

| **Sample** | **Space group** | **α (^o^)** | **β (^o^)** | **γ (^o^)** | **a (Å)** | **b (Å)** | **c (Å)** | **V (Å3)** |
| --- | --- | --- | --- | --- | --- | --- | --- | --- |
| **Fe_7_Se_8_** | P 31 | 90 | 90 | 120 | 7.17 | 7.17 | 17.46 | 777.344 |


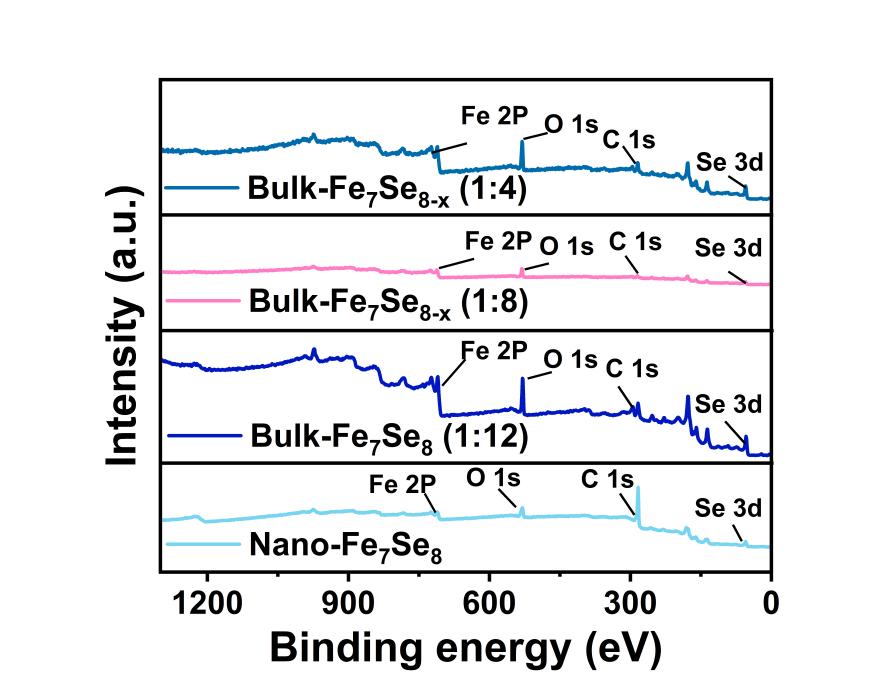


**Figure S1.** XPS survey spectra of three samples.


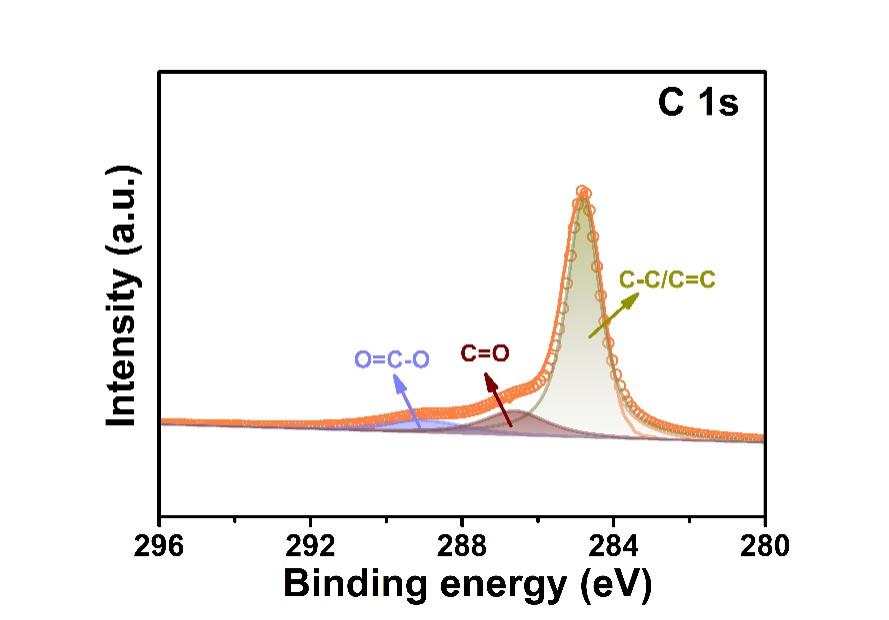


**Figure S2.** XPS spectrum of C 1s for nano-Fe_7_Se_8_.

**Table S2.** Element contents of three samples from ICP-AES results.

| **Sample** | **Measured element** | **Percent (%)** | **Atom (%)** |
| --- | --- | --- | --- |
| **Bulk-Fe_7_Se_8-x_（1: 4）** | Fe | 40.7228 | 7 |
|  | Se | 40.5482 | 6.97 |
| **Bulk-Fe_7_Se_8-x_（1: 8）** | Fe | 40.6103 | 7 |
|  | Se | 59.1999 | 7.22 |
| **Bulk-Fe_7_Se_8_ （1: 12）** | Fe | 38.3651 | 7 |
|  | Se | 61.5611 | 7.95 |
| **Nano-Fe_7_Se_8_** | Fe | 16.9096 | 7 |
|  | Se | 27.4139 | 8.02 |





**Figure S3.** (a, b) SEM images of bulk-Fe_7_Se_8-x_ (1: 8).


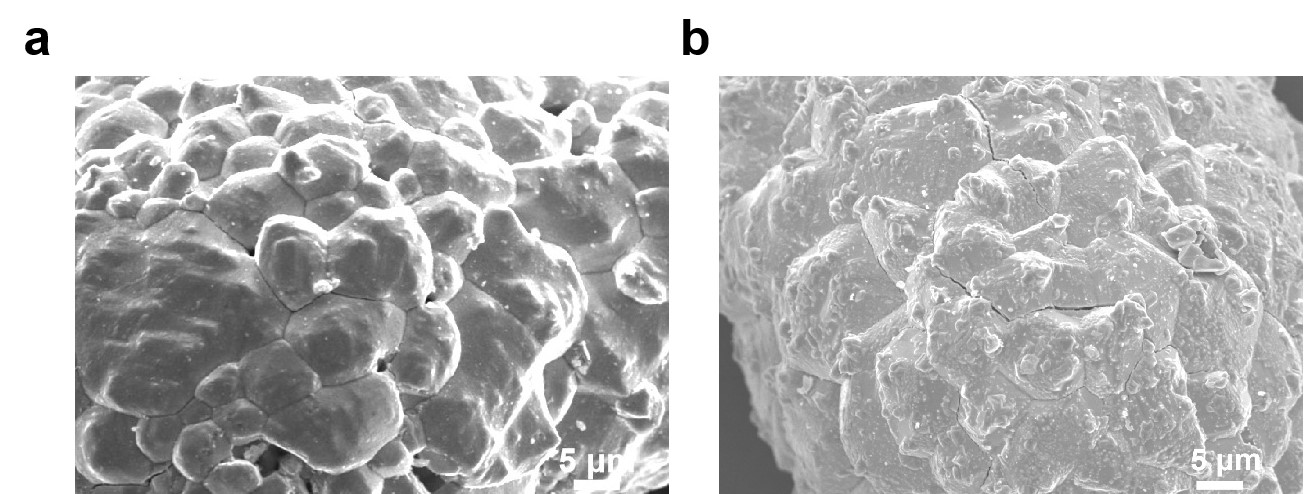


**Figure S4.** (a) and (b) SEM images of bulk-Fe_7_Se_8-x_ (1: 4) and bulk-Fe_7_Se_8_ (1: 12).

**
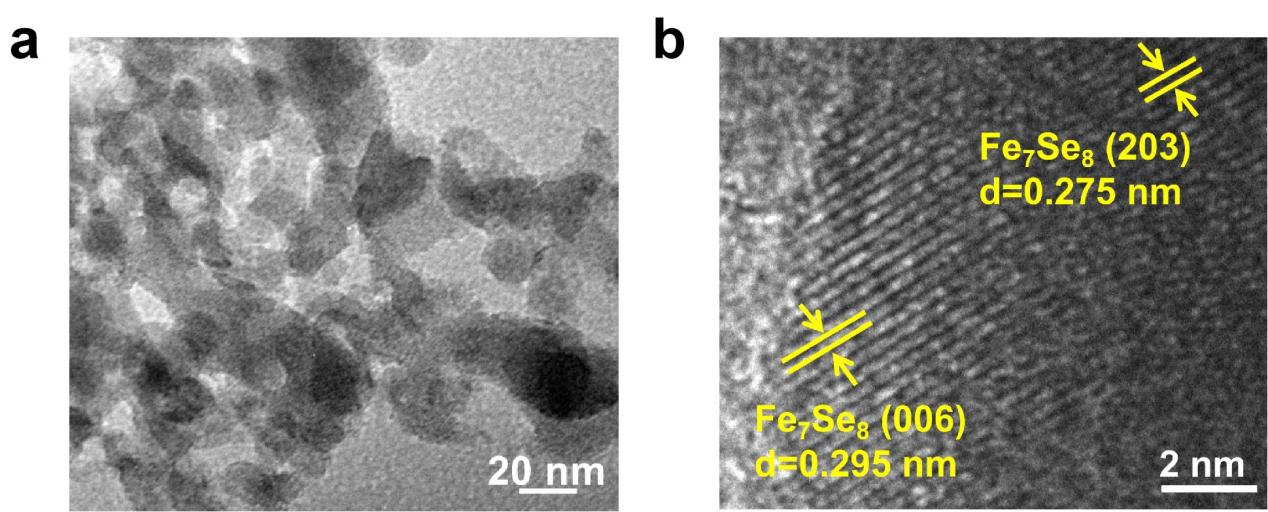
**

**Figure S5.** (a) and (b) TEM images of nano-Fe_7_Se_8_.


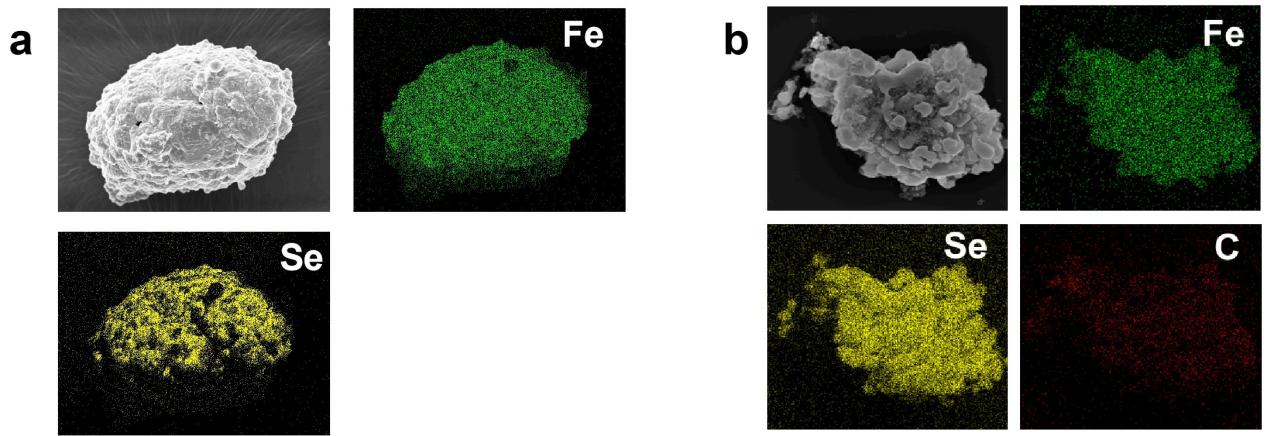


**Figure S6.** (a,b) Element mappings of bulk-Fe_7_Se_8_ (1: 12) and nano-Fe_7_Se_8_.


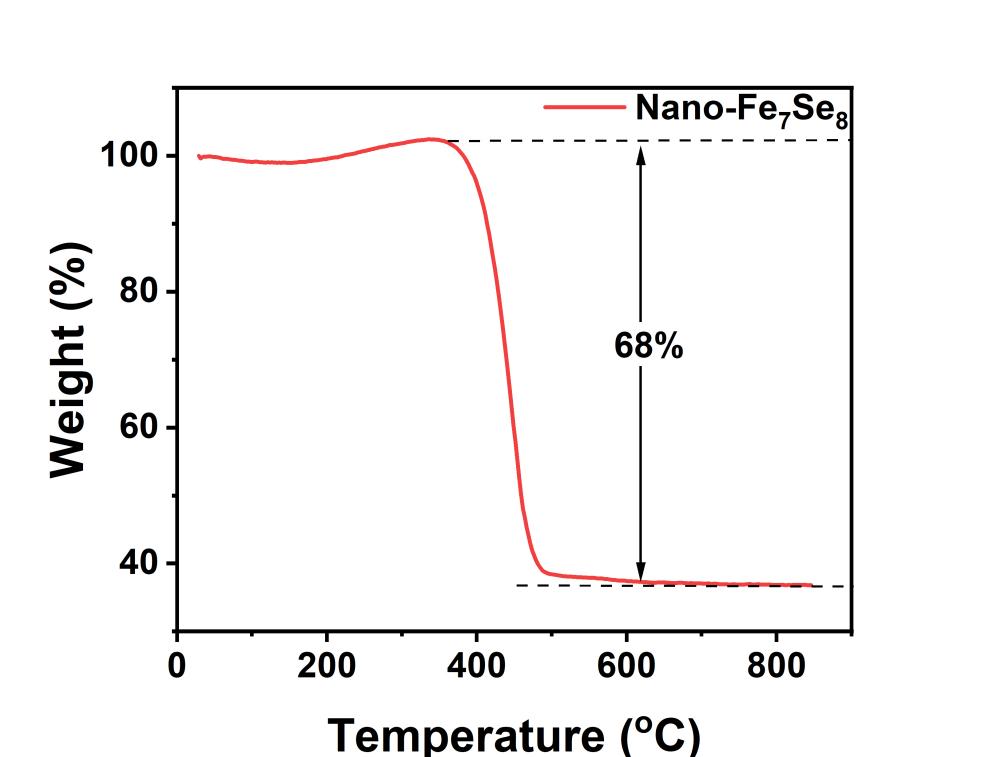


**Figure S7.** TGA curve of nano-Fe_7_Se_8_.


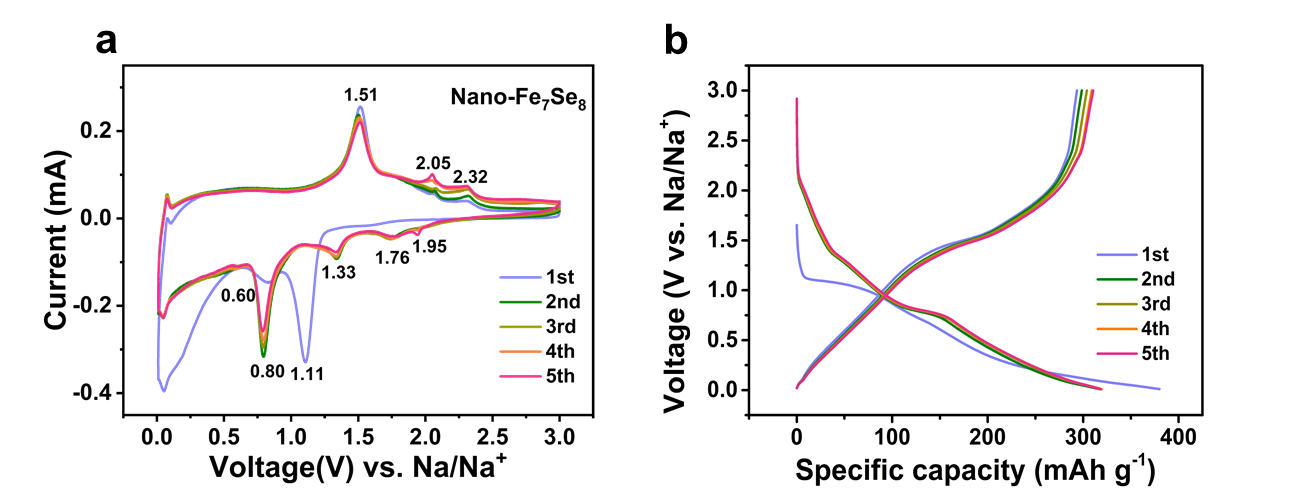


**Figure S8.** (a) CV curves at 0.1 mV s^-1^, and (b) discharge/charge profiles for the first five cycles at 0.5 A g^-1^ of nano-Fe_7_Se_8_.


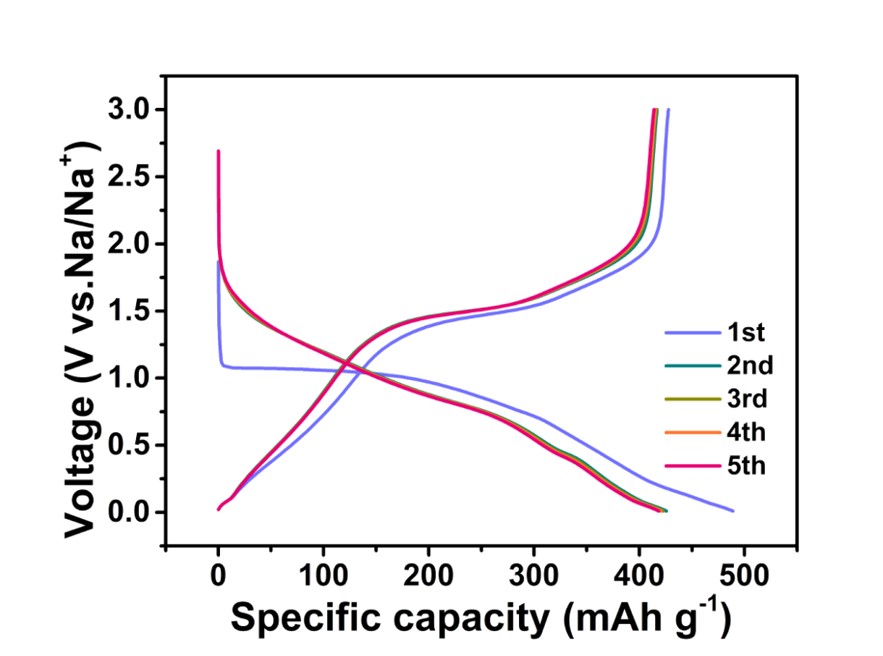


**Figure S9.** Discharge/charge profiles for the first five cycles at 0.5 A g^-1^ of bulk-Fe_7_Se_8-x_ (1: 8).

**Table S3.** Sodium storage performances comparison of Fe_7_Se_8_ related composites.

| **Sample name** | **Current density**  **（A g^-1^）** | **Cycle number** | **Specific capacity（mAh g^-1^）** | **Reference** |
| --- | --- | --- | --- | --- |
| Fe_7_Se_8_/N-CNFs | 1 | 2000 | 340.8 | [9] |
| Fe_7_Se_8_ NRBs | 2 | 1800 | 250 | [10] |
| Fe_7_Se_8_@C/N NBs | 1 | 1000 | 345.2 | [11] |
| Fe_7_Se_8_@NC | 5 | 6000 | 185.6 | [12] |
| Fe_7_Se_8_@NC | 1 | 1200 | 339 | [13] |
| Fe_7_Se_8_@C | 3 | 500 | 218 | [14] |
| Fe_7_Se_8_@C | 2 | 720 | 319 | [15] |
| bulk-Fe_7_Se_8-x_ | 5 | 1300 | 384 | This work |


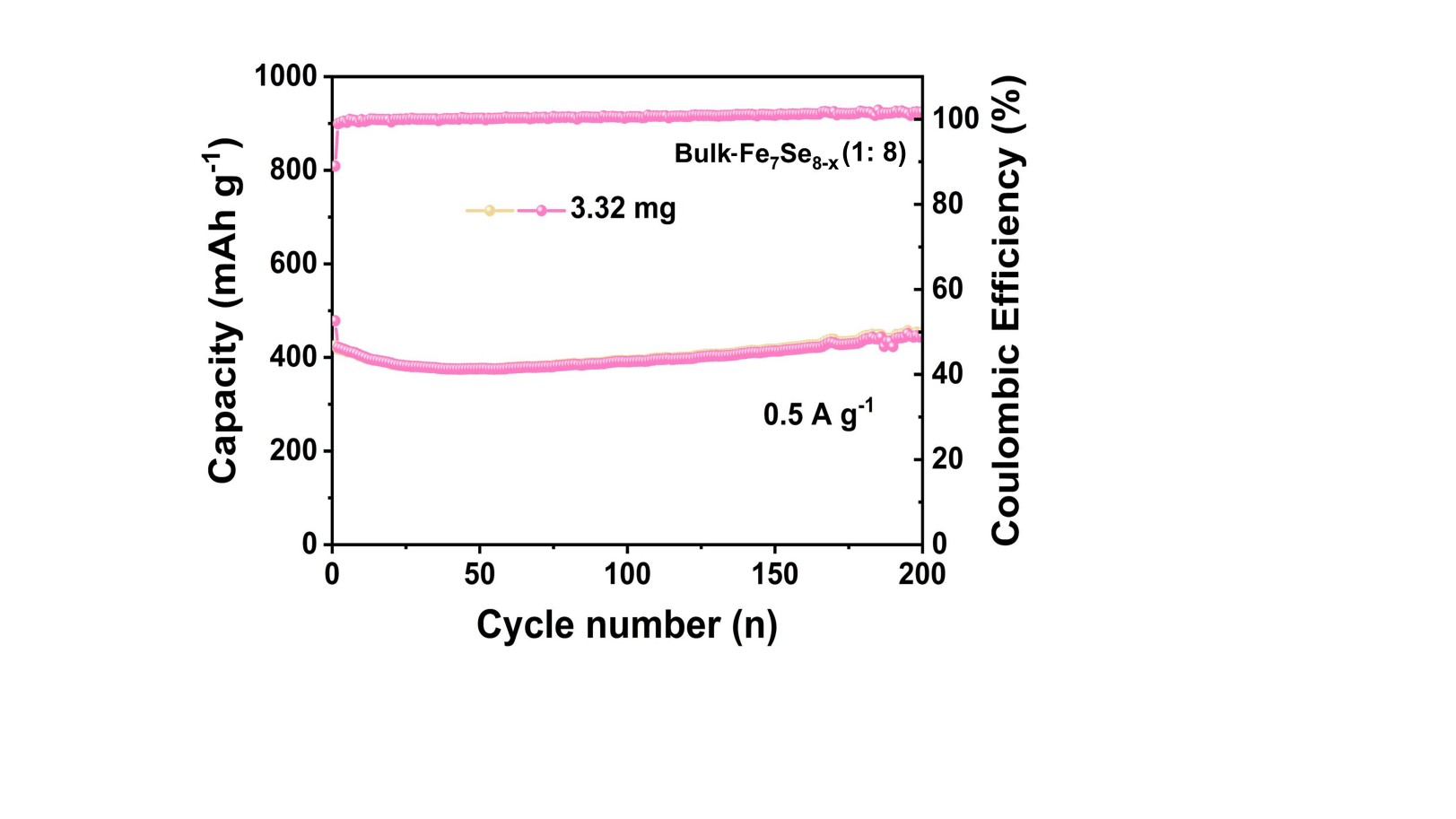


**Figure S10.** Cyclic performance of bulk-Fe_7_Se_8-x_ (1: 8) with a high mass loading at 0.5 A g^-1^.


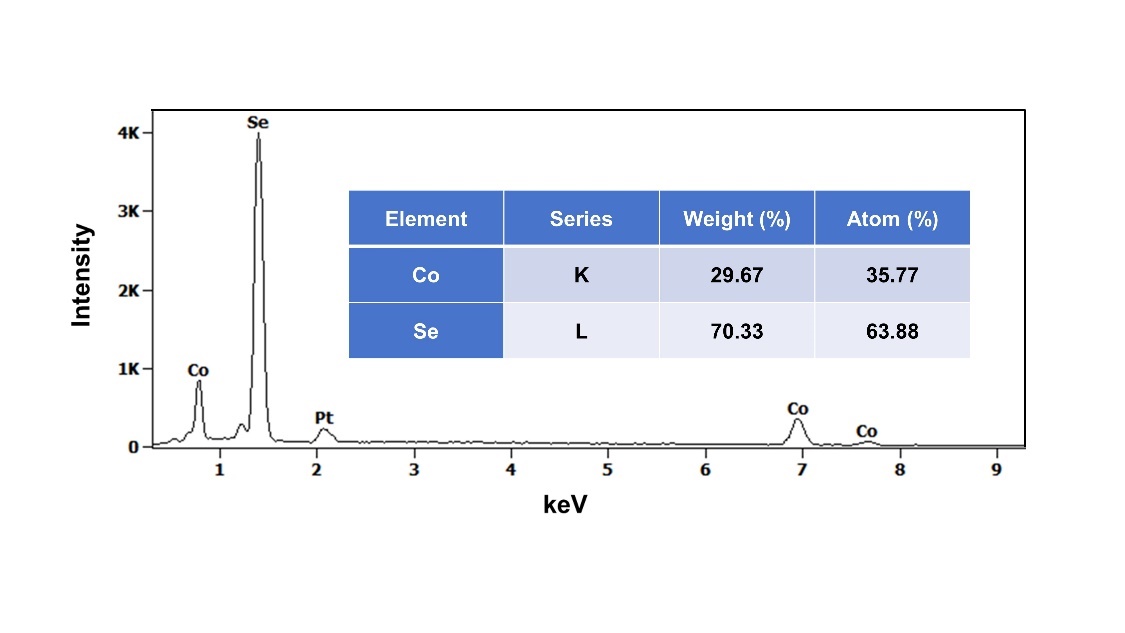


**Figure S11.** EDS spectrum of CoSe_2-x_ (the inset is the atomic ratio of Co and Se).


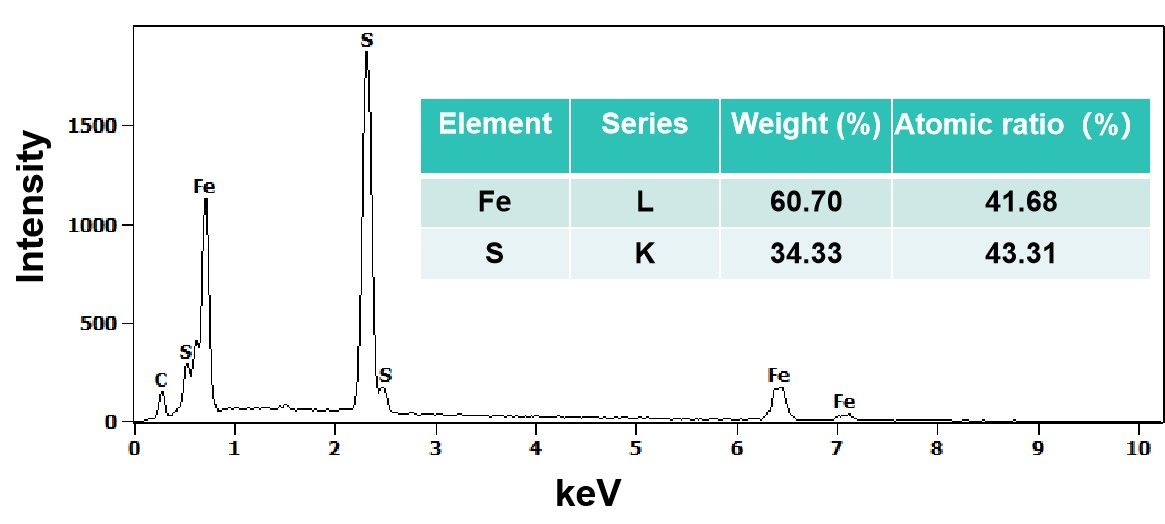


**Figure S12.** EDS spectrum of Fe_7_S_8-x_ (the inset is the atomic ratio of Fe and S).


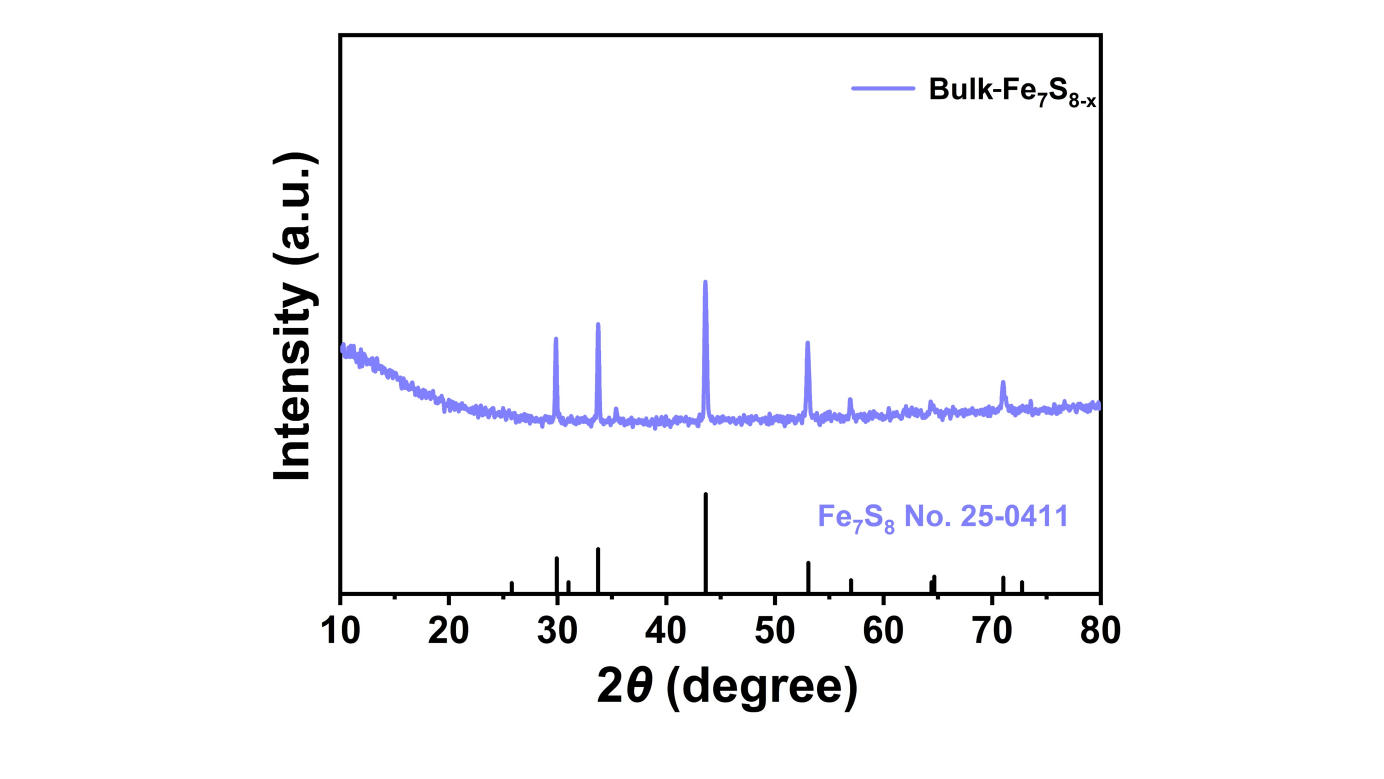


**Figure S13.** XRD pattern of bulk-Fe_7_S_8-x_.


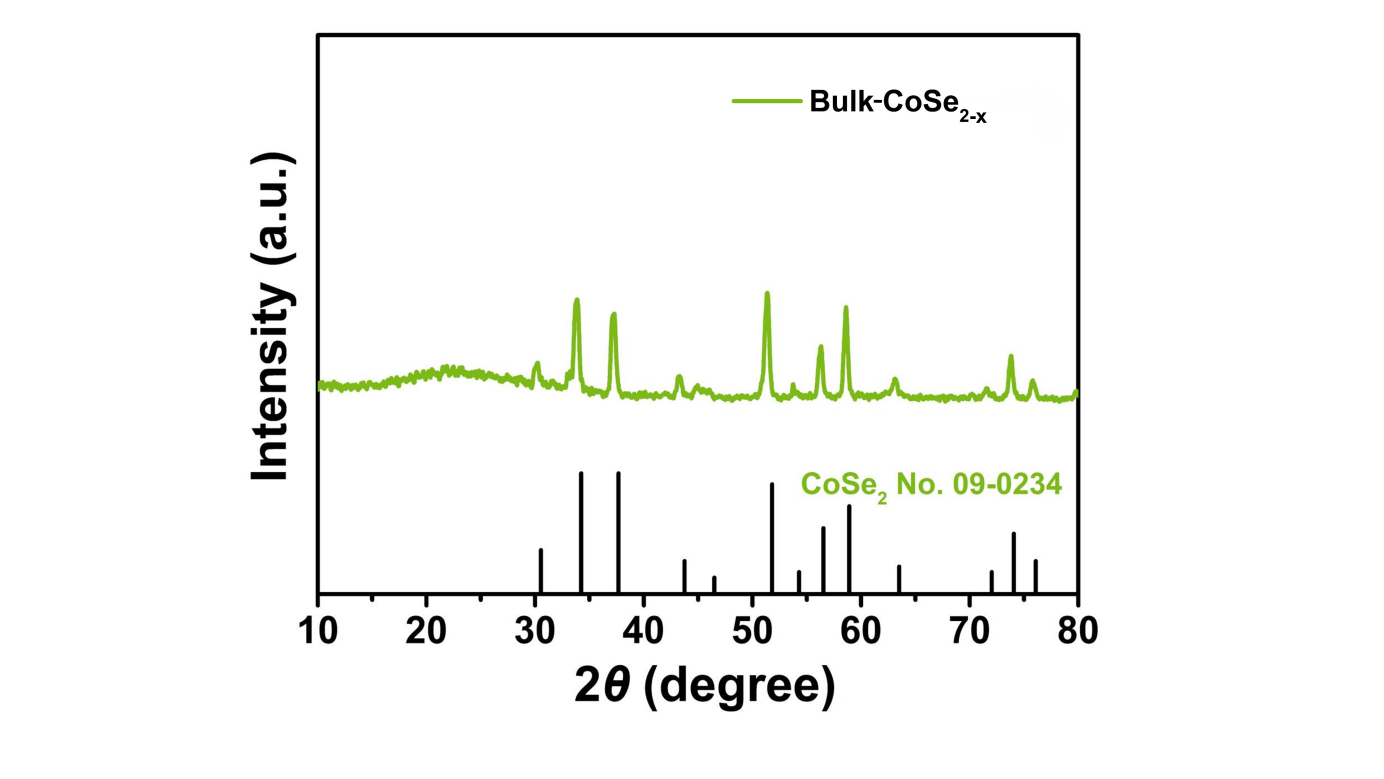


**Figure S14.** XRD pattern of bulk-CoSe_2-x_.


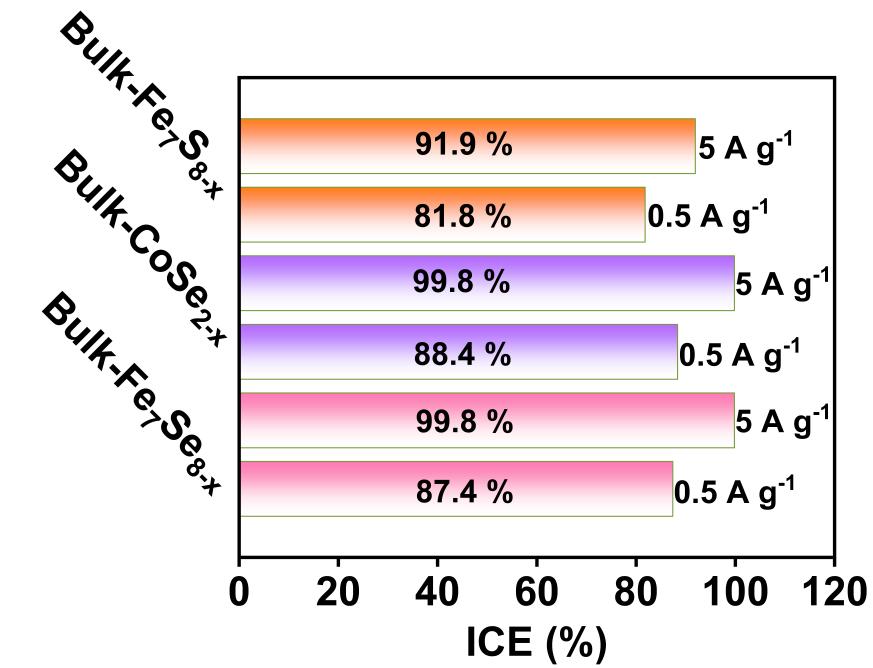


**Figure S15.** ICE values of three electrodes.


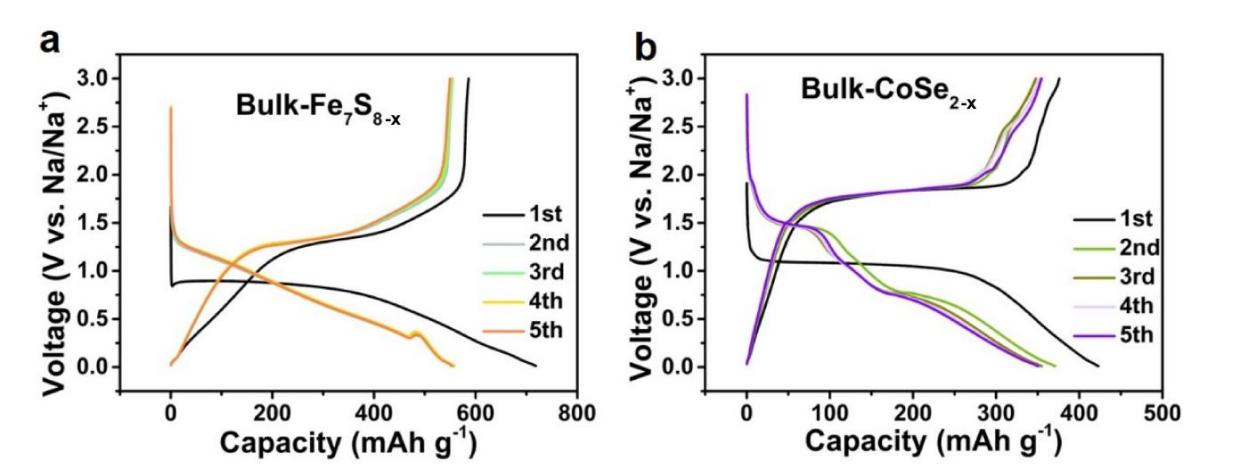


**Figure S16.** Discharge/charge profiles for the first five cycles at 0.5 A g^-1^ of bulk-Fe_7_Se_8-x_ (a) and bulk-CoSe_2-x_(b).


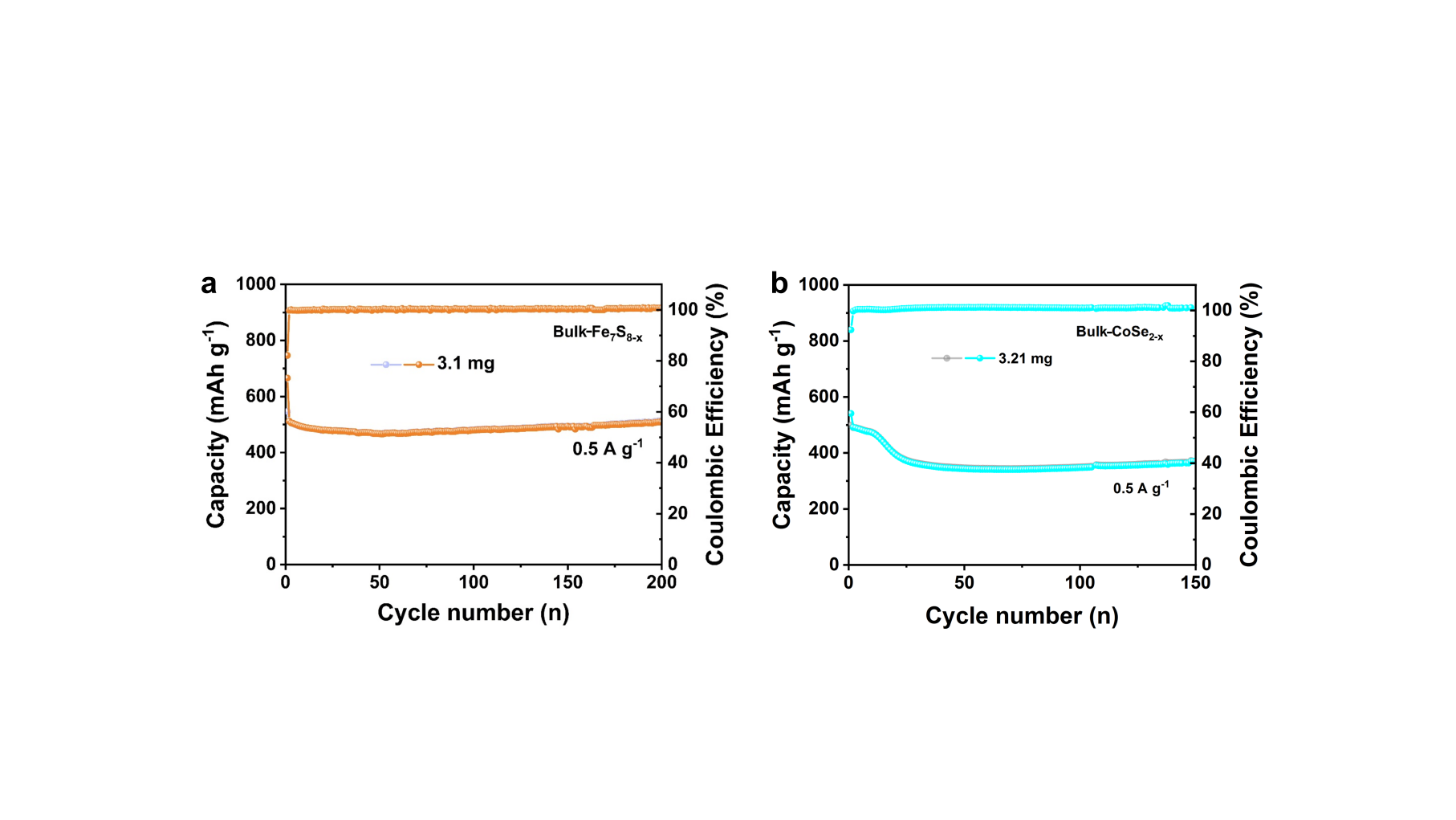


**Figure S17.** Cyclic performances of (a) bulk-Fe_7_S_8-x_, and (b) bulk-CoSe_2-x_ with high mass loadings at 0.5 A g^-1^.


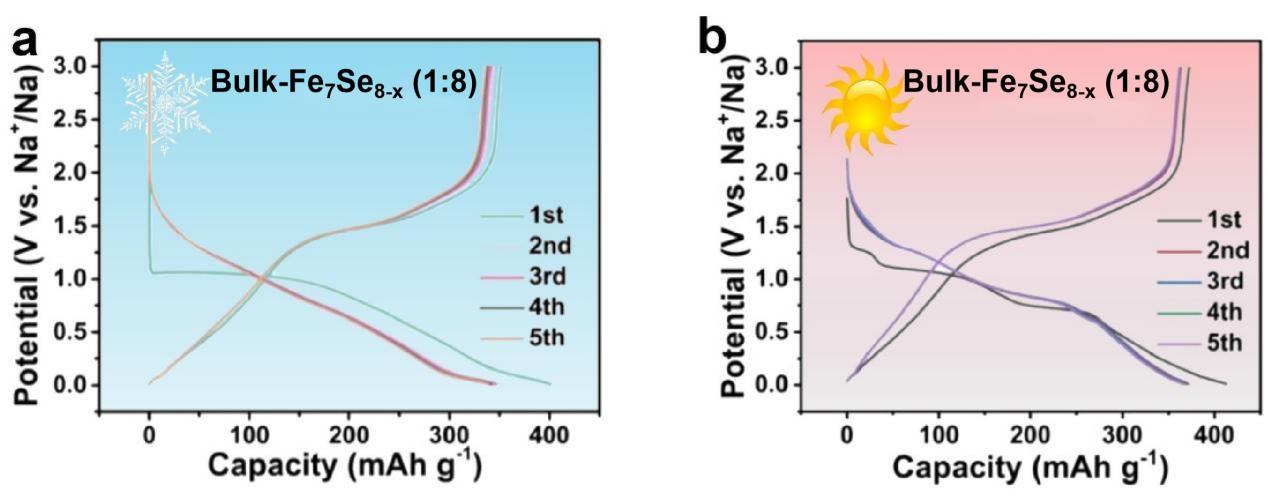


**Figure S18.** Discharge/charge profiles of bulk-Fe_7_Se_8-x_ (1: 8) at 0 °C (a) and 40 °C (b).





**Figure S19.** *In-situ* XRD patterns of bulk-Fe_7_Se_8-x_.


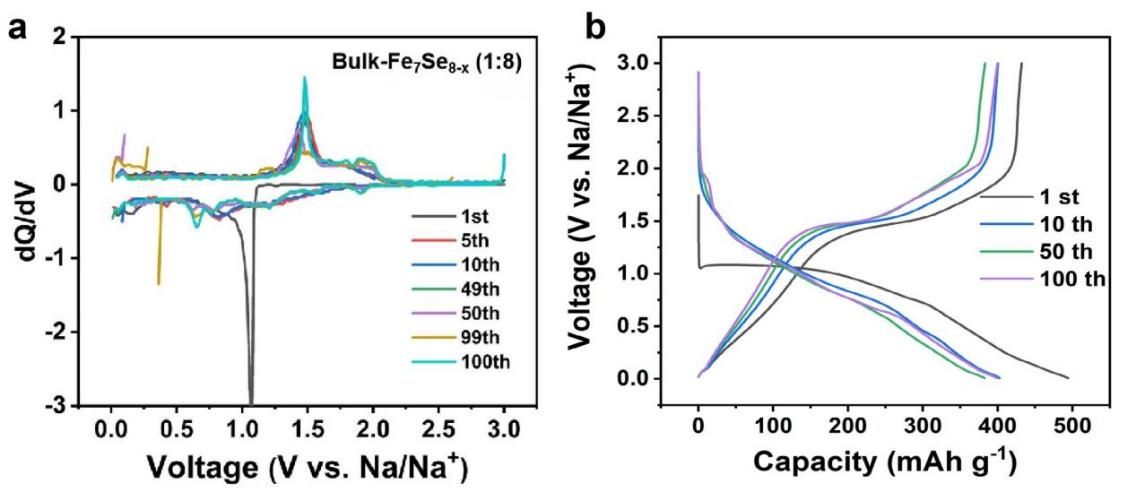


**Figure S20.** (a) Derivative plots (dQ/dV) and (b) discharge/charge profiles of bulk-Fe_7_Se_8-x_ (1: 8).


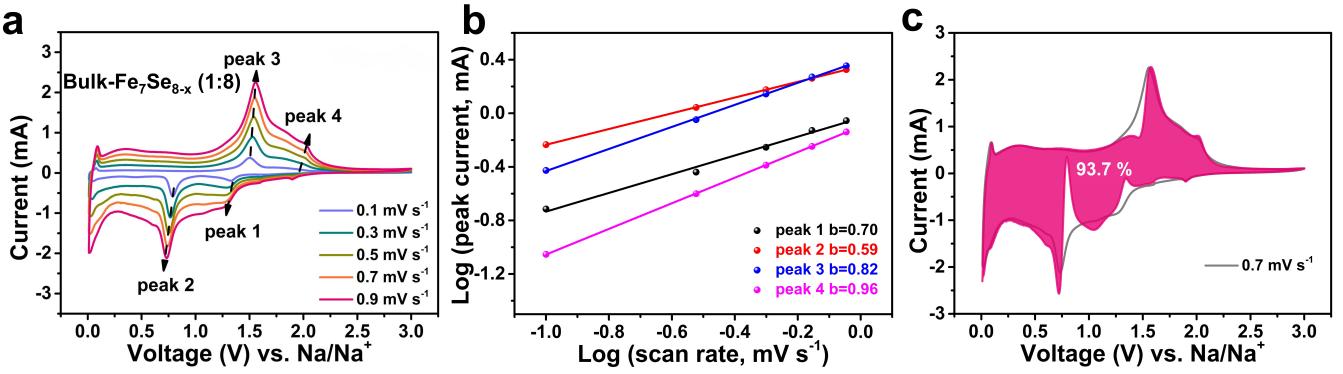


**Figure S21.** Kinetics analysis of bulk-Fe_7_Se_8-x_ (1: 8) : (a) CV curves at different scan rates, (b) relationship between log i and log v, (c) capacitance contribution to the charge storage at 0.7 mV s^-1^.

**Table S4** Capacitance contribution ratios of three electrodes.

| **Scan Rate (mV s^-1^)** | **Bulk-Fe_7_Se_8-x_ (1: 8)** | **Bulk-F_e7_Se_8_ (1: 12)** | **Nano-Fe_7_Se_8_** |
| --- | --- | --- | --- |
| 0.1 | 87.7% | 58.9% | 57.8% |
| 0.3 | 89.8% | 70.1% | 69.3% |
| 0.5 | 91.1% | 81.7% | 72.3% |
| 0.7 | 93.7% | 82.9% | 78.7% |
| 0.9 | 95.0% | 90.2% | 82.1% |


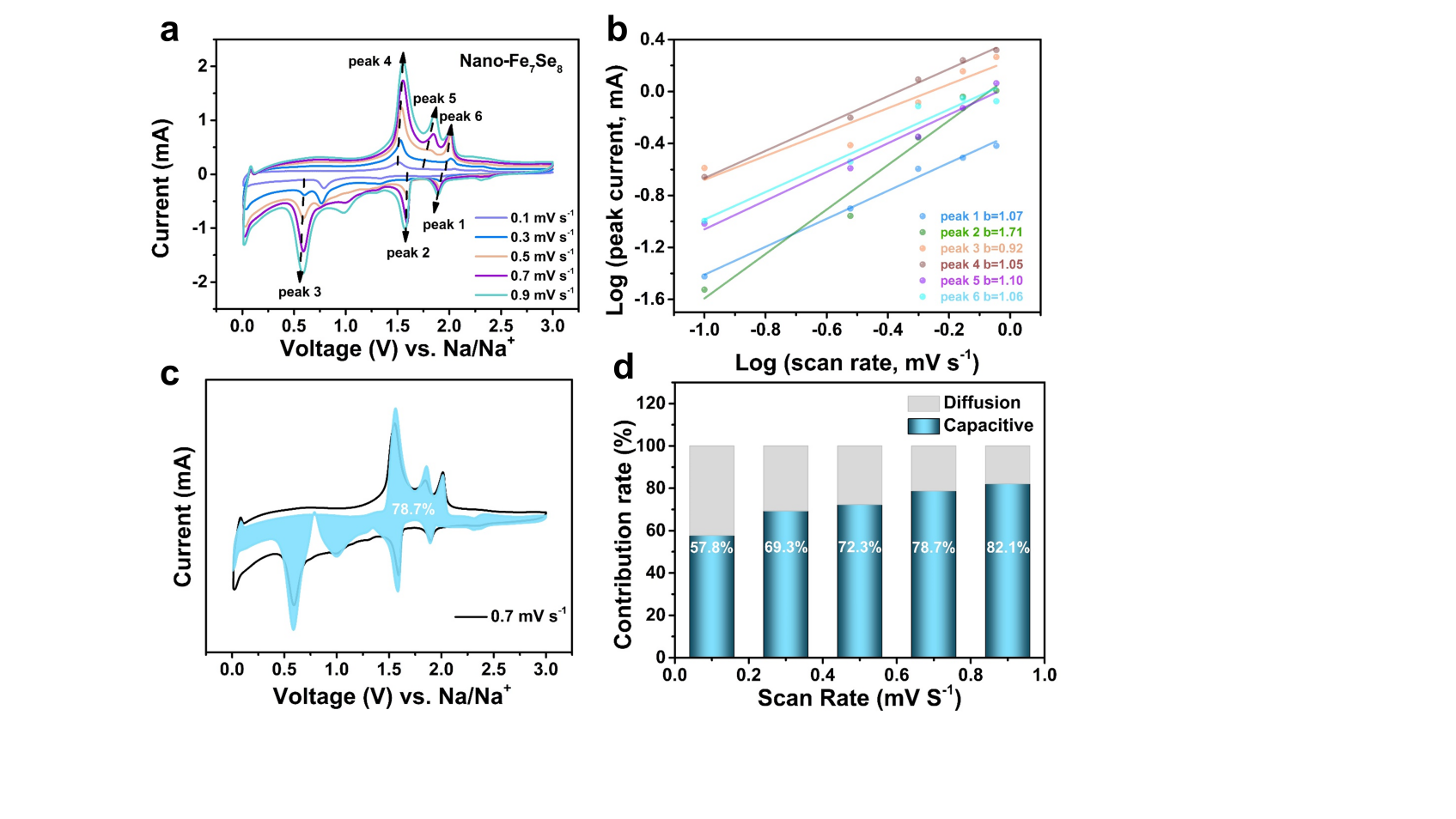


**Figure S22.** Kinetics analysis of nano-Fe_7_Se_8_: (a) CV curves at different scan rates, (b) relationship between log i and log v, (c) capacitance contribution to the charge storage at 0.7 mV s^-1^ and (d) capacitance contribution ratios at different scan rates.


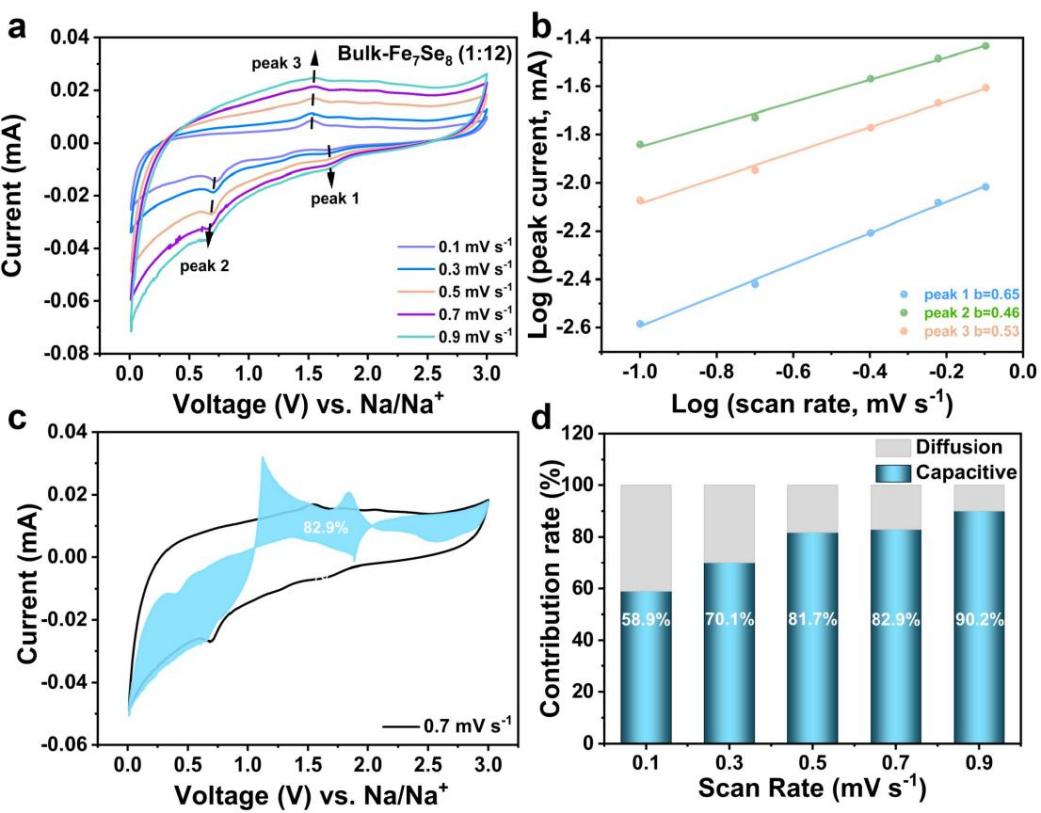


**Figure S23.** Kinetics analysis of bulk-Fe_7_Se_8_ (1: 12) : (a) CV curves at different scan rates, (b) relationship between log i and log v, (c) capacitance contribution to the charge storage at 0.7 mV s^-1^, and (d) capacitance contribution ratios at different scan rates.

**Table S5** Reaction impedance derived from EIS spectra.

| **Sample name** | **R_s_ (Ω)** | **R_ct_ (Ω)** |
| --- | --- | --- |
| Bulk-Fe_7_Se_8-x_ (1: 8) | 4.65 | 1.43 |
| Bulk-Fe_7_Se_8_ (1: 12) | 3.80 | 2.00 |
| Nano-Fe_7_Se_8_ | 5.14 | 5.30 |


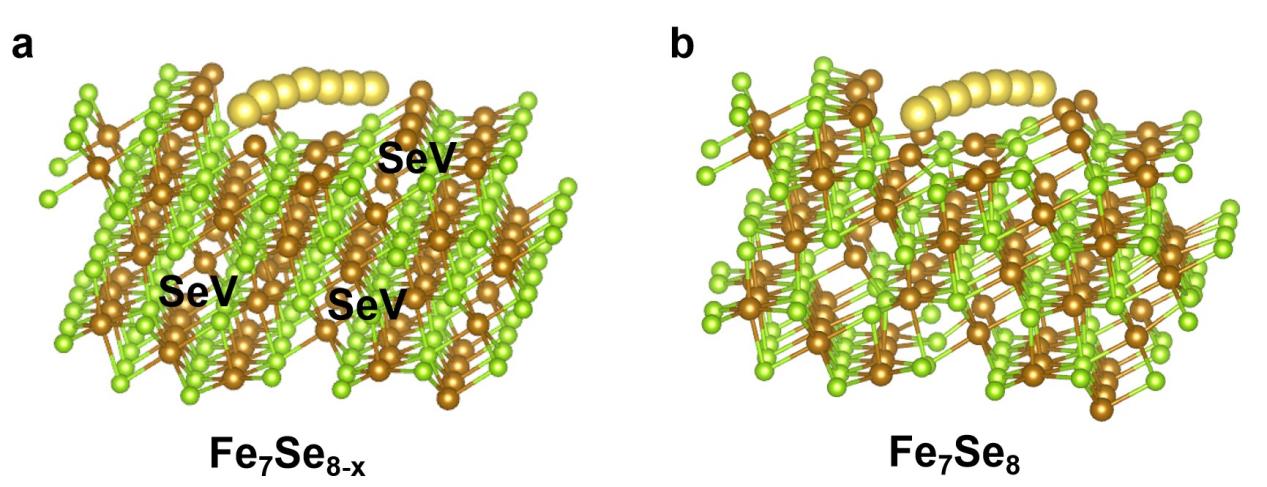


**Figure S24.** Na^+^ migration paths of Fe_7_Se_8-x_ and Fe_7_Se_8_.

| **Sample** | **Measured element** | **Percent (%)** | **Atom (%)** | **Cycles** |
| --- | --- | --- | --- | --- |
| **Bulk-Fe_7_Se_8-x_（1:4）** | Fe | 53.2725 | 7 | 50 |
|  | Se | 46.7275 | 6.14 |  |
| **Bulk-Fe_7_Se_8-x_（1:8）** | Fe | 52.7506 | 7 | 50 |
|  | Se | 47.2494 | 6.27 |  |
| **Bulk-Fe_7_Se_8_（1:12）** | Fe | 60.5536 | 7 | 50 |
|  | Se | 39.4464 | 4.56 |  |
| **Bulk-Fe_7_Se_8-x_（1:8）** | Fe | 57.4713 | 7 | 100 |
|  | Se | 42.5287 | 5.18 |  |

**Table S6.** Element contents of samples from EDS results.


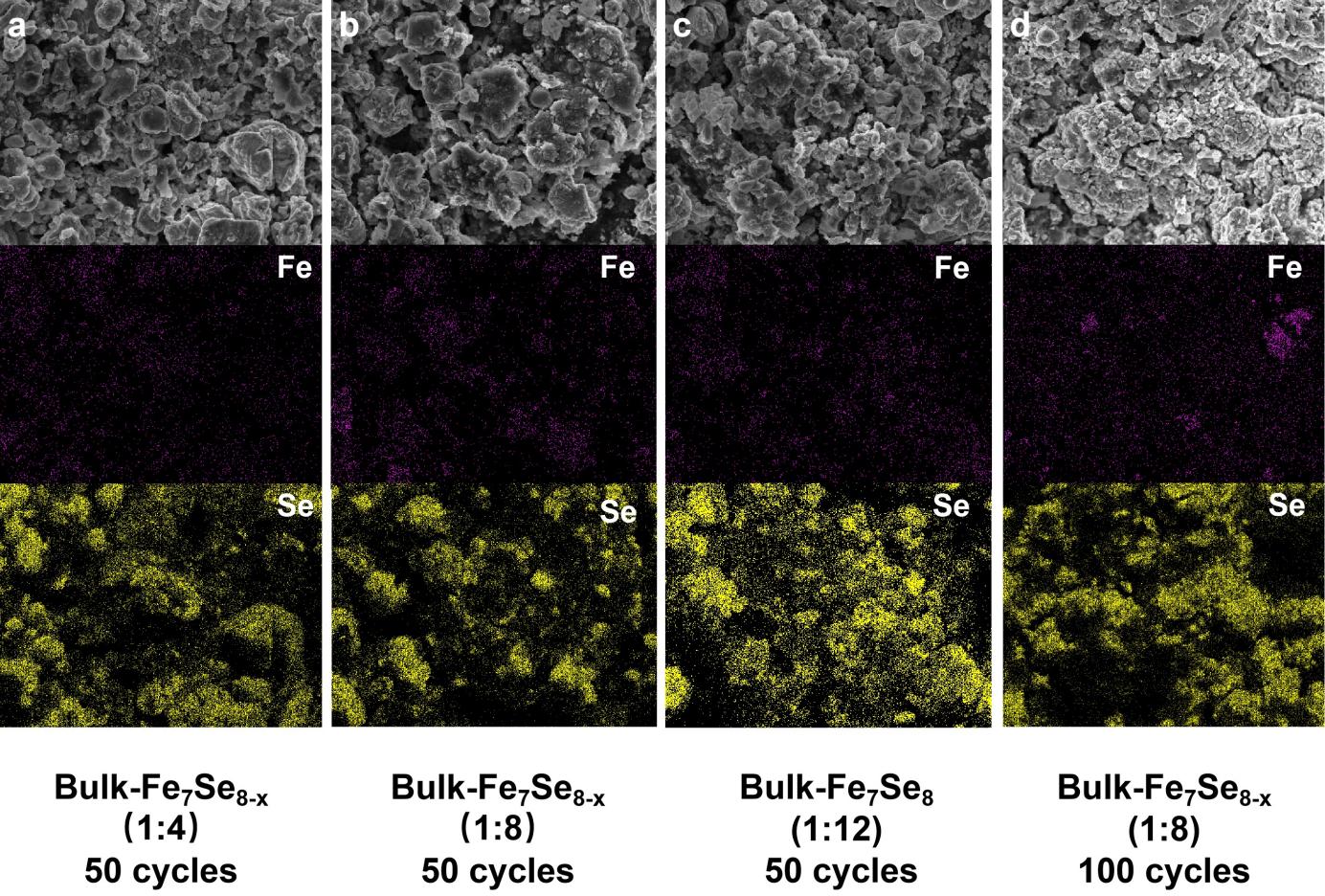


**Figure S25.** Element mappings of bulk-Fe_7_Se_8-x_ (1: 4), bulk-Fe_7_Se_8-x_ (1: 8) and bulk-Fe_7_Se_8_ (1: 12).


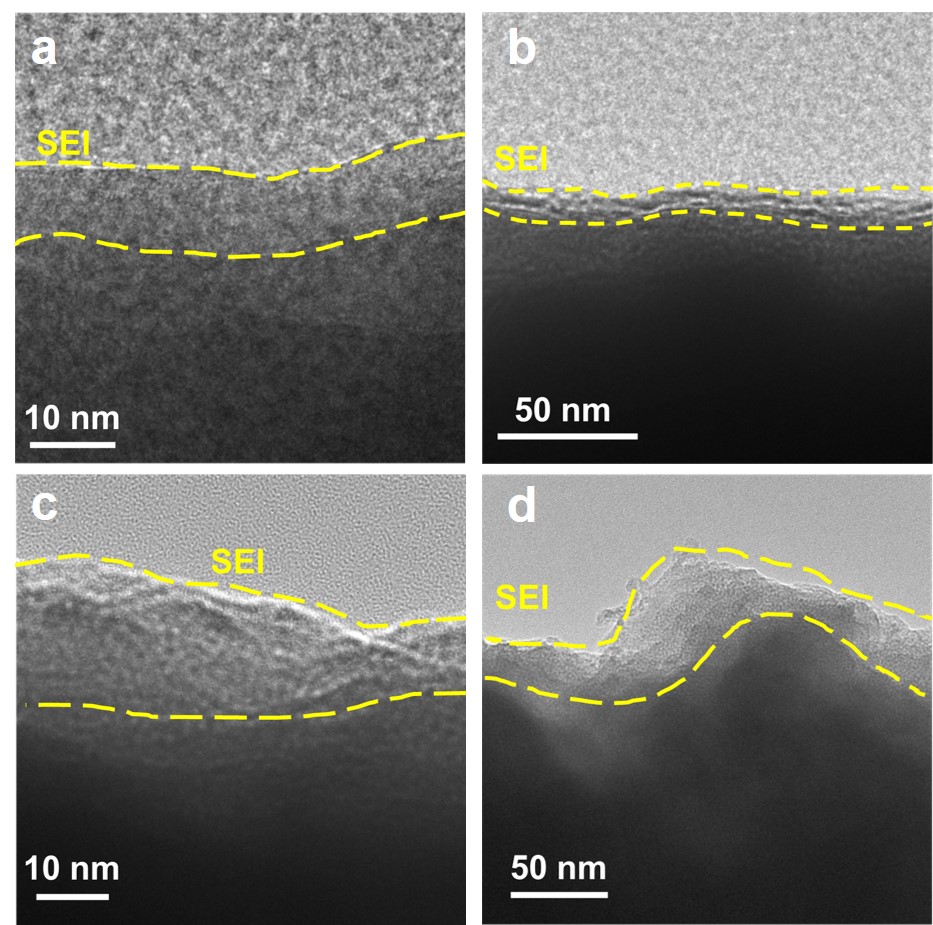


**Figure S26.** HRTEM images of bulk-Fe_7_Se_8-x_ (1: 8).


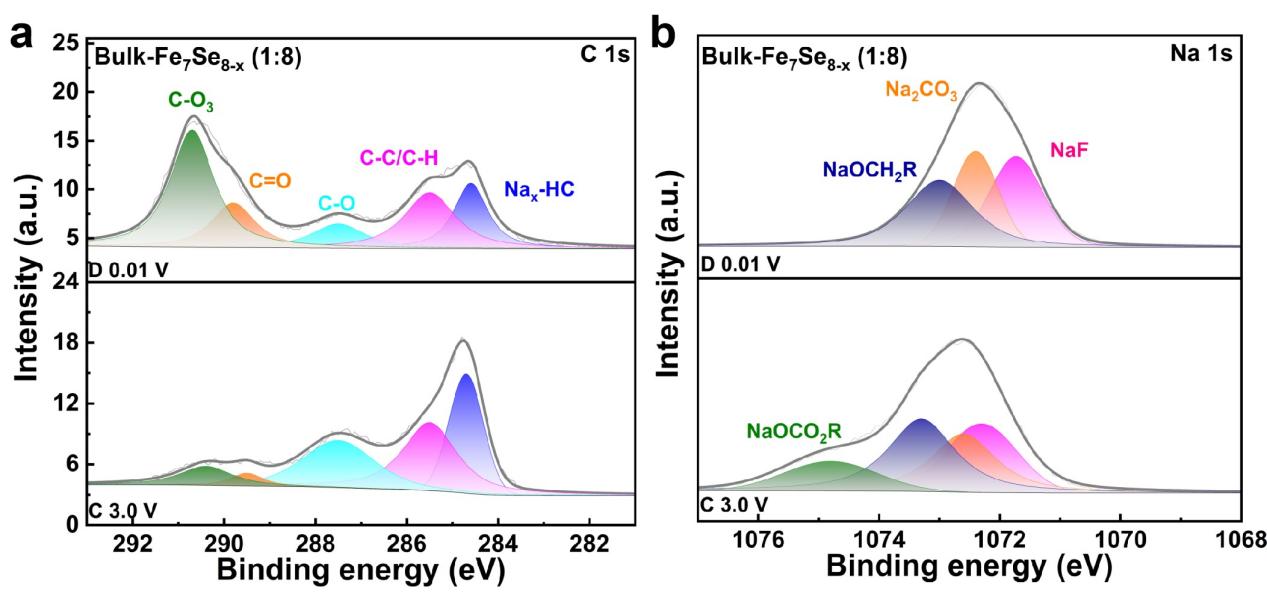


**Figure S27.** *Ex-situ* XPS spectra of bulk-Fe_7_Se_8-x_ (1: 8) electrodes at different discharge/charge states for the first cycle: (a) C 1s, and (b) Na 1s.


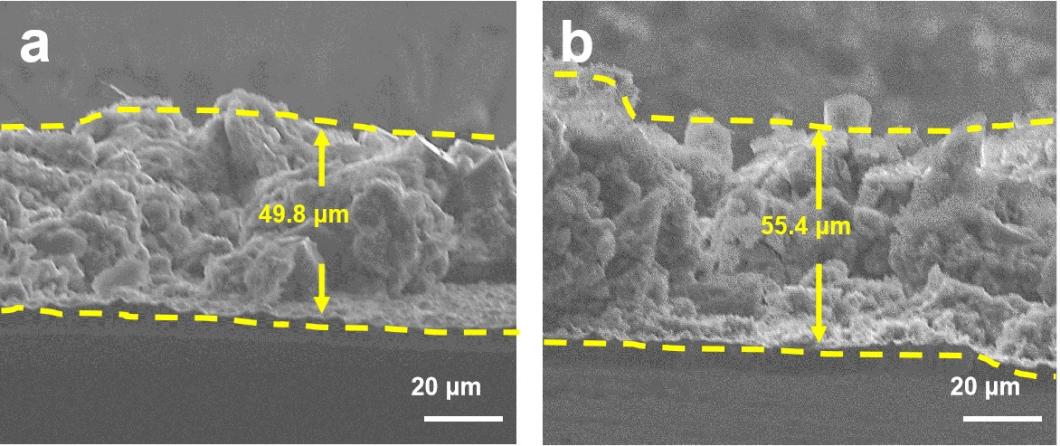


**Figure S28.** SEM cross-section images of bulk-Fe_7_Se_8-x_ (1: 8) : (a) Initial electrode, (b) 10 cycles.


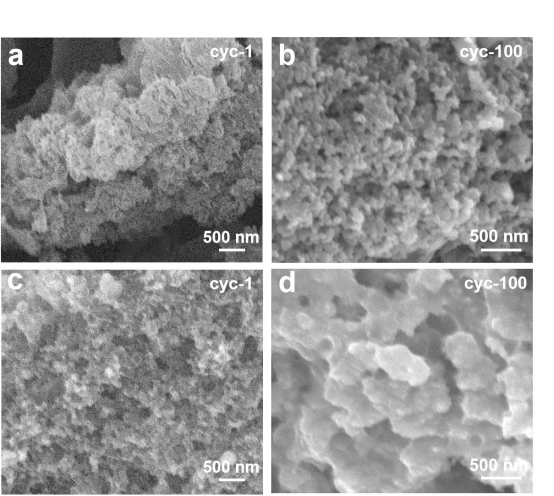


**Figure S29.** SEM images of bulk-Fe_7_Se_8-x_ (1: 8) : (a) Initial electrode, and (b) 100 cycles.


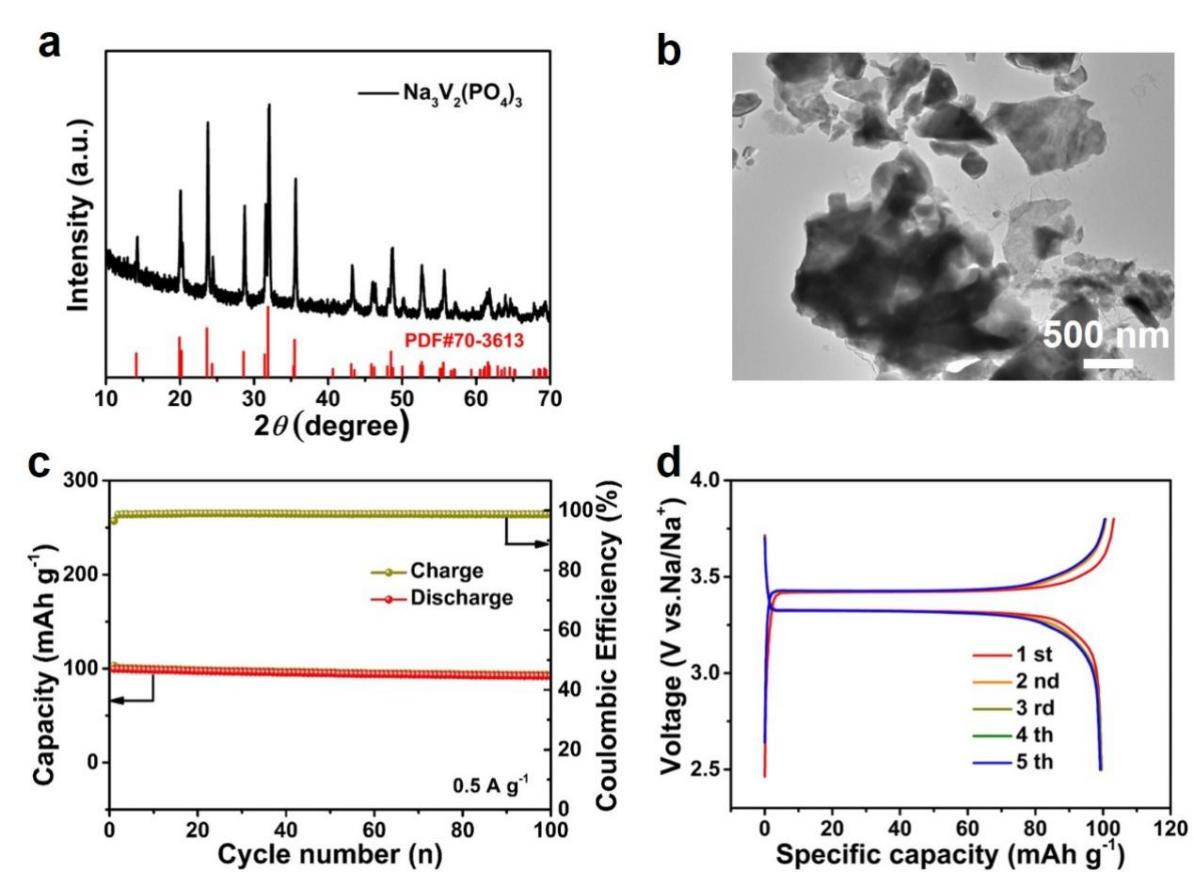


**Figure S30.** (a) XRD pattern, (b) TEM image, (c) cyclic performance at 0.5 A g^-1^, and (d) charge/discharge profiles at 0.5 A g^-1^ of Na_3_V_2_(PO_4_)_3_@rGO.


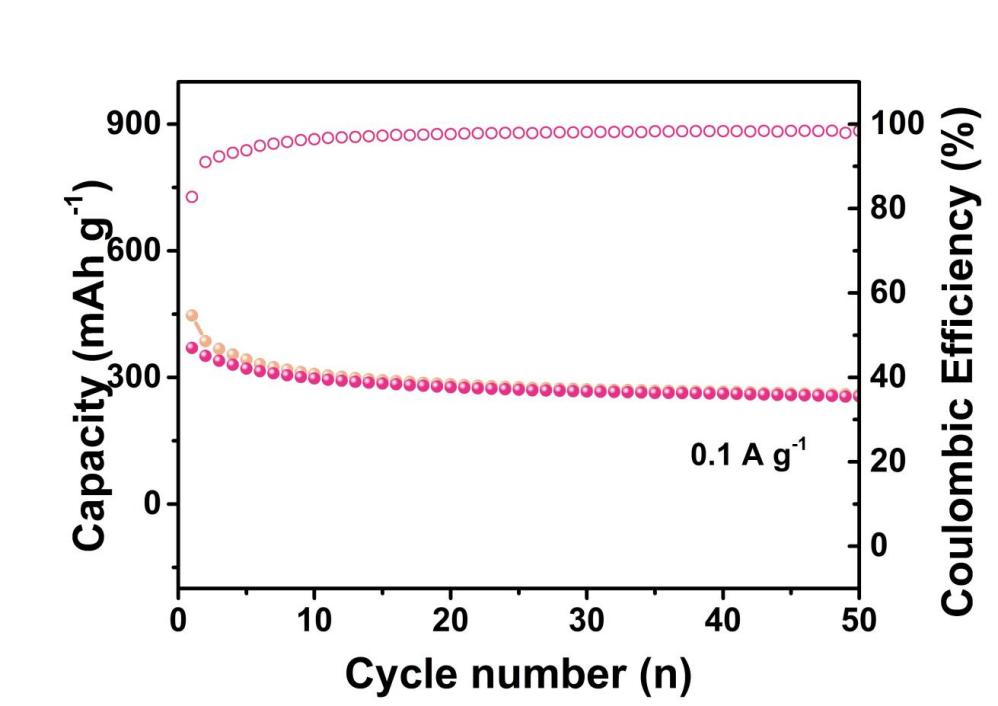


**Figure S31.** Cyclic performance of Na_3_V_2_(PO_4_)_3_@rGO//bulk-Fe_7_Se_8-x_ full cell at 0.1 A g^-1^.

**References**

1. Xu, Y.; Wei, Q.; Xu, C.; Li, Q.; An, Q.; Zhang, P.; Sheng, J.; Zhou, L.; Mai, L. “Layer‐by‐Layer Na_3_V_2_(PO_4_)_3_ Embedded in Reduced Graphene Oxide as Superior Rate and Ultralong‐Life Sodium‐Ion Battery Cathode,” *Adv. Energy Mater.* 6 (2016) 1600389.
2. Wang, M.; Peng, A.; Jiang, J.; Zeng, M.; Yang, Z.; Chen, J.; Guo, B.; Ma, Z.; Yu, B.; Zhang, Y.; Li, X. “Heterointerface Synergistic Na^+^ Storage Fundamental Mechanism for CoSeO_3_ Playing as Anode for Sodium Ion Batteries/Capacitors,” *Chem. Eng. J.* 433 (2022) 123456.
3. Jiang, M.; Hu, Y.; Mao, B.; Wang, Y.; Yang, Z.; Meng, T.; Wang, X.; Cao, M. “Strain-Regulated Gibbs Free Energy Enables Reversible Redox Chemistry of Chalcogenides for Sodium Ion Batteries,” *Nat. Commun.* 13 (2022) 5588.
4. Hu, X.J.; Zhu, R.Y.; Wang, B.B.; Wang, H.; Liu, X.Y. “Sn Catalyst for Efficient Reversible Conversion Between MoSe_2_ and Mo/Na_2_Se for High-Performance Energy Storage,” *Chem. Eng. J.* 440 (2022) 135819.
5. Brezesinski, T.; Wang, J.; Tolbert, S.H.; Dunn, B. “Ordered Mesoporous Alpha-MoO3 with Iso-Oriented Nanocrystalline Walls for Thin-Film Pseudocapacitors,” *Nat. Mater.* 9 (2010) 146-51.
6. Kresse, G.; Furthmüller, J. “Efficient Iterative Schemes for ab Initio Total-Energy Calculations Using a Plane-Wave Basis Set,” *Physical Review B* 54 (1996) 11169-11186.
7. Kresse, G.; Furthmüller, J. “Efficiency of Ab-Initio Total Energy Calculations for Metals and Semiconductors Using a Plane-Wave Basis Set Comput,” *Mater. Sci* 6 (1996) 15-50.
8. Perdew, J. P.; Burke, K.; Ernzerhof, M. “Generalized Gradient Approximation Made Simple,” *Phys. Rev. Lett.*  24 (2020) 439-449.
9. Zhang, D.M.; Jia, J.H.; Yang, C.C.; Jiang, Q. Fe_7_Se_8_ Nanoparticles Anchored on N-Doped Carbon Nanofibers as High-Rate Anode for Sodium-Ion Batteries. *Energy Storage Mater.* 24 (2020) 439-449.
10. Tian, W.Z.; Ma, W.Z.; Feng, Z.Y.; Tian, F.; Li, H.B.; Liu, J.; Xiong, S.L. “Formation of Hierarchical Fe_7_Se_8_ Nanorod Bundles with Enhanced Sodium Storage Properties,” *J. Energy Chem.* 44 (2020) 97-105.
11. Sun, Z.H.; Wu, X.L.; Gu, Z.Y.; Han, P.; Zhao, B.L.; Qu, D.Y.; Gao, L.F.; Liu, Z.M.; Han, D.X.; Niu, L. “Rationally Designed Nitrogen-Doped Yolk-Shell Fe_7_Se_8_/Carbon Nanoboxes with Enhanced Sodium Storage in Half/Full Cells,” *Carbon* 166 (2020) 175-182.
12. Yang, S.; Jiang, J.M.; He, W.J.; Wu, L.Y.; Xu, Y.H.; Ding, B.; Dou, H.; Zhang, X.G. “Nitrogen-Doped Carbon Encapsulating Fe_7_Se_8_ Anode with Core-Shell Structure Enables High-Performance Sodium-Ion Capacitors,” *J. Colloid Interf. Sci.* 630 (2023) 144-154.
13. Wan, M.; Zeng, R.; Chen, K.; Liu, G.; Chen, W.; Wang, L.; Zhang, N.; Xue, L.; Zhang, W.; Huang, Y. “Fe_7_Se_8_ Nanoparticles Encapsulated by Nitrogen-Doped Carbon with High Sodium Storage Performance and Evolving Redox Reactions,” *Energy Storage Mater.* 10 (2018) 114-121.
14. Xu, X.J.; Liu, J.; Liu, J.W.; Ouyang, L.Z.; Hu, R.Z.; Wang, H.; Yang, L.C.; Zhu, M. “A General Metal‐Organic Framework (MOF)‐Derived Selenidation Strategy for In Situ Carbon‐Encapsulated Metal Selenides as High‐Rate Anodes for Na‐Ion Batteries,” *Adv. Funct. Mater.* 28 (2018) 1707573.
15. Yuan, J.J.; Gan, Y.F.; Xu, X.J.; Mu, M.Q.; He, H.S.; Li, X.K.; Zhang, X.K.; Liu, J. “Construction of Fe_7_Se_8_@Carbon Nanotubes with Enhanced Sodium/Potassium Storage,” *J. Colloid Interf. Sci.* 626 (2022) 355-363.
